# Bioactive aporphines and flavonoids from a fermented beverage target metabolic inflammatory pathways in obesity and type 2 diabetes

**DOI:** 10.1038/s41598-025-30778-9

**Published:** 2025-12-08

**Authors:** Xiurong Wu, Yang Qiu, Rui Dai, Zixu Huang, Jinghan Wang, Xiantao Yan, Xiangzhen Nie, Ronghan Liu

**Affiliations:** 1 School of Food and Health, Guilin Tourism University, Guilin, China; 2 School of Life Sciences and Food Engineering, Hanshan Normal University, Chaozhou, China; 3 Key Laboratory of Industrialized Processing and Safety of Guangxi Cuisine, Guilin Tourism University, Education Department of Guangxi Zhuang Autonomous Region, Guilin, China; 4 Guangxi Engineering Research Center for Large-Scale Preparation & Nutrients and Hygiene of Guangxi Cuisine, Guilin, China

**Keywords:** Functional beverage, Obesity, Type 2 diabetes, Aporphines, Flavonoids, Molecular dynamic simulation, Biochemistry, Computational biology and bioinformatics, Drug discovery

## Abstract

The global syndemic of obesity and type 2 diabetes (T2D) demands safe, multi-targeted dietary interventions. FH03FS, a sterilized fermented beverage blended from five medicinal food homologous (MFH) plants, represents a promising candidate. However, the phytochemical profile and mechanistic basis for its potential efficacy remain uncharacterized. Ultra-performance liquid chromatography-tandem mass spectrometry (UPLC-MS/MS) analysis of FH03FS revealed a wide spectrum of compounds. Ten key bioactive ingredients, primarily aporphines and flavonoids, were identified as the primary active components based on favorable pharmacokinetic properties. In silico Absorption, Distribution, Metabolism, Excretion, and Toxicity (ADMET) profiling indicated high gastrointestinal (GI) absorption and low toxicity risks for these compounds. Network pharmacology demonstrated the modulation of core pathways driving metabolic inflammation. Molecular docking preliminarily identified multiple high-affinity interactions between the bioactive compounds and core targets. The exceptional stability and strong binding of representative complexes, notably Morin-ESR1 and Asimilobine-PPARG, were further validated by molecular dynamics (MD) simulations. This study demonstrates that FH03FS, rich in aporphines and flavonoids, exerts multi-target effects against obesity and T2D. Our findings thereby provide a phytochemical and mechanistic foundation for its development as a ready-to-consume functional beverage and offer testable hypotheses for future validation.

**Supplementary Information:**

The online version contains supplementary material available at 10.1038/s41598-025-30778-9.

## Introduction

 The dual epidemics of diabetes and obesity are increasingly recognized as a major global health crisis, characterized by intertwined metabolic dysfunction and chronic low-grade inflammation. This complex pathophysiology often undermines conventional single-target therapeutic approaches^1^. While drugs like glucagon-like peptide-1 (GLP-1) agonists and sodium-glucose cotransporter 2 (SGLT2) inhibitors demonstrate clinical efficacy, they often exhibit prohibitive costs, adverse side effects, and limited capacity to address comorbid pathologies^2,3^. These limitations highlight an urgent need for safe, affordable, and accessible multi-target dietary strategies capable of simultaneously modulating metabolic-inflammatory networks.

FH03FS is a novel sterilized fermented beverage developed within the framework of Traditional Chinese Medicine (TCM) principles for addressing metabolic dysregulation. TCM historically attributes such conditions to patterns like “phlegm-dampness” and “spleen-stomach dysfunction”, which manifest clinically as obesity, insulin resistance, and chronic low-grade inflammation^4,5^. This beverage integrates five medicinal food homologous (MFH) herbs with documented complementary bioactivities:

Radix of *Millettia speciosa* (Niudali) is traditionally used to reinforce *Qi* (a concept correlating with energy metabolism and vitality). It alleviates obesity and glycolipid disorders by stimulating adipose thermogenesis and activating the IRS2/PI3K-Akt/GLUT4 pathway^6,7^.

Lotus leaf (Heye) is traditionally employed to dispel dampness and clear heat^4^ (concepts associated with resolving metabolic inflammation and dyslipidemia). It ameliorates hyperglycemia and obesity by inhibiting α-glucosidase, enhancing thermogenesis via the β3-AR/AMPK/p38 pathway, and remodeling gut microbiota^8,9^.

Monk fruit (Luohanguo) is traditionally used to moisten the lungs and relieve cough^4^ (concepts associated with airway protection and systemic metabolic homeostasis). It serves as a beneficial sweetener and alleviates diabetic liver lipid disorders via modulation of hepatic metabolic pathways^10^.

Tangerine peel (Chenpi) is traditionally used to regulate *Qi* and resolve phlegm^4^ (concepts associated with digestive health and metabolic inflammation). It ameliorates diabetic vasculopathy by improving endothelial function, suppressing vascular inflammation, and activating the AMPK pathway^11^.

*Cinnamomi Cortex* (Rougui) is traditionally utilized to warm meridians and promote circulation (concepts associated with improving microcirculation and insulin sensitivity). It ameliorates hyperglycemia and dyslipidemia by inhibiting digestive enzymes, regulating hepatic glucose production, and modulating cholesterol uptake^12^.

Probiotic fermentation is a well-established strategy to enhance the bioactivity and bioavailability of phytochemicals while improving the organoleptic properties of food products^13,14^. However, terminal sterilization, which is critical for safety and shelf-stability, presents a paradoxical challenge, as thermal processing may degrade heat-sensitive compounds or alter bioactive structures^15,16^. This creates a significant disconnect between studying raw herbs or unsterilized ferments and the actual consumed product.

Consequently, a critical translational gap persists in current research. While prior studies have predominantly focused on profiling the raw substrates or unsterilized ferments, the phytochemical composition and, crucially, the potential bioactivity of the sterilized final product remain largely uninvestigated. This oversight is significant, as the final sterilization step may alter the bioactive profile, thereby determining the actual efficacy of the commercial product. The direct relevance of prior findings to the sterilized, ready-to-consume beverage therefore remains uncertain, impeding the rational development of reproducible and efficacious functional beverages.

To address this gap, our study focuses exclusively on the finalized, sterilized product FH03FS. First, we employed Ultra-performance liquid chromatography-tandem mass spectrometry (UPLC-MS/MS) to identify and characterize the chemical constituents in FH03FS. We then integrated network pharmacology to predict the multi-target mechanisms through which its active constituents might concurrently combat obesity and T2D. Furthermore, molecular docking and molecular dynamics (MD) simulations were employed to validate the predicted interactions between representative compounds and core targets at the atomic level. This work aims to bridge the aforementioned translational gap by providing essential phytochemical data and mechanistic hypotheses directly relevant to the sterilized product. It thereby establishes a foundation for developing FH03FS bioactivity and informs the design of functional beverage targeting metabolic health.

## Materials & methods

### Materials, microorganisms, and reagents

The four MFH materials were purchased from Beijing Tongrentang Health Pharmaceutical Co., Ltd. (Beijing, China), including: Lotus leaf, Monk fruit, Tangerine peel, and *Cinnamomi Cortex*. Radix of *Millettia speciosa* was obtained from Guangxi Yiyan Biotechnology Co., Ltd. (Batch No. 2023005, Fangchenggang, China).

The probiotic strains *Lacticaseibacillus paracasei* CS01 (CGMCC No. 31418) and *Lactiplantibacillus plantarum* CS02 (CGMCC No. 31419) were isolated and characterized by our research team, with preservation and quality control conducted by the China General Microbiological Culture Collection Center (CGMCC, Beijing, China).

UPLC-MS/MS analysis was performed using Liquid chromatography-mass spectrometry (LC-MS)-grade solvents, including methanol and formic acid (Thermo Fisher Scientific, United States) and ultrapure water (Merck, Germany).

**Fig. 1:**
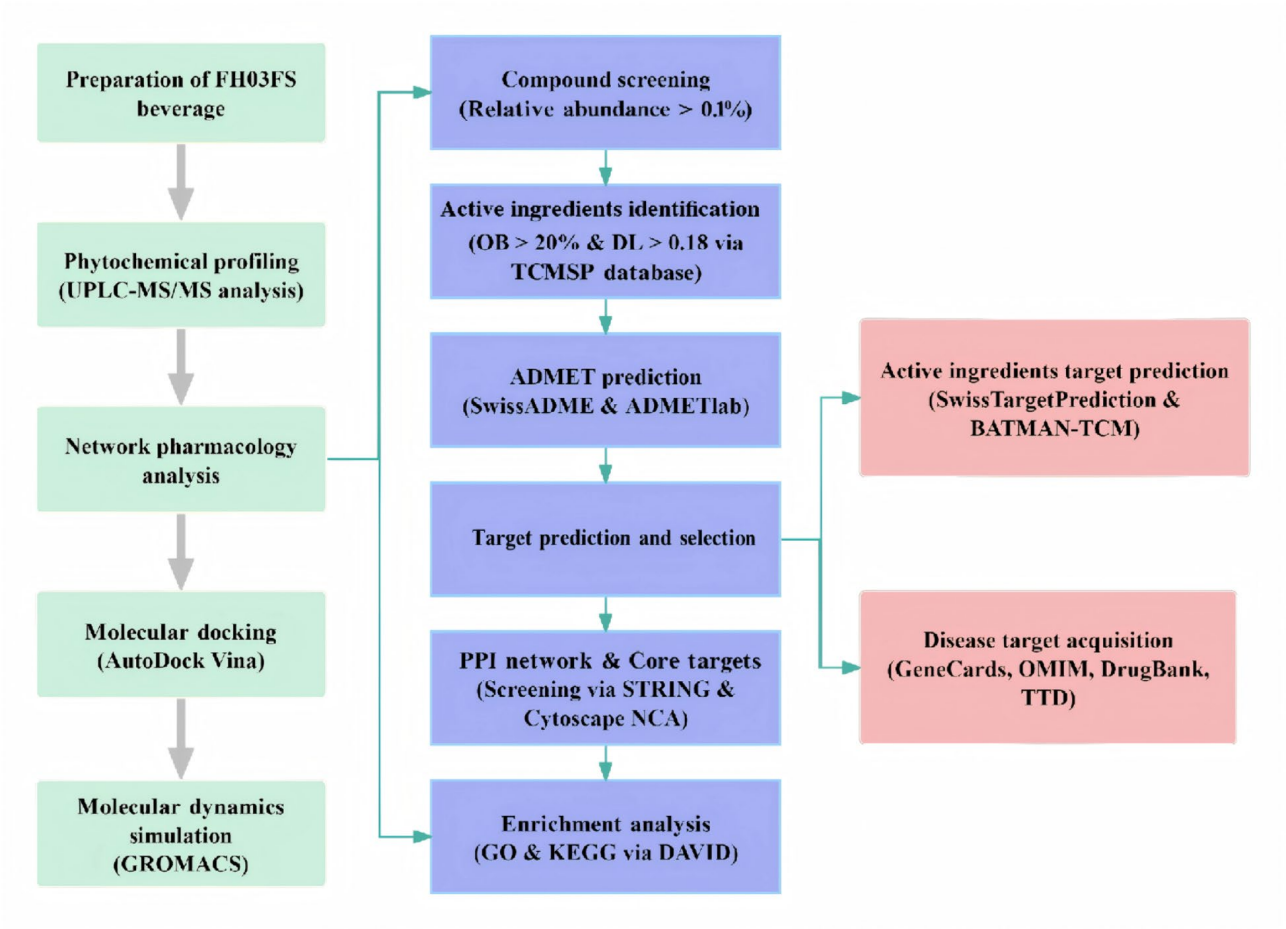
Workflow of the integrated strategy to investigate the phytochemical profile and mechanisms of FH03FS against obesity and type 2 diabetes (T2D).

The overall experimental strategy of this study is summarized in Fig. 1. Briefly, our integrated approach combined phytochemical profiling, network pharmacology analysis, and computational validation to elucidate the multi-target mechanisms of FH03FS against obesity and T2D.

### Preparation of FH03FS

The FH03FS preparation method was modified from previous studies^16,17^. Briefly, five MFH materials mentioned above were pulverized to powder at predefined mass ratios^17^. The powder blend was suspended in purified water at a 1:15 w/v ratio with 30-minute maceration, followed by sterilizing at 121 ℃ for 15 min, then centrifuged at 10,000 × g for 1 min. The supernatant was inoculated with activated *Lacticaseibacillus paracasei* CS01 and *Lactiplantibacillus plantarum* CS02 (1:1, 3% v/v, 1–5 × 10⁷ CFU/mL) and cultured at 37 ℃ for 45 h, followed by pasteurization (80 ℃, 15 min) to obtain FH03FS. Three independent production batches were stored at − 80 ℃. For each batch, one aliquot was randomly selected and analyzed in triplicate (*n* = 3 technical replicates per treatment).

### Phytochemical profiling

#### Compound extraction

The extraction procedure followed the reported method^18^. The samples (100 µL) were transferred to EP tubes and resuspended in prechilled 80% methanol by vortexing. After incubation on ice for 5 min, the mixtures were centrifuged at 15,000 × g (4 ℃, 20 min). Some of the supernatant was diluted with LC-MS-grade water to 53% (v/v) methanol and recentrifuged under identical conditions. Finally, the supernatant was injected into the UPLC-MS/MS system for analysis. Equal extract volumes were combined as quality control (QC) samples.

#### UPLC-MS/MS conditions

UPLC-MS/MS analyses were performed on a Vanquish UPLC system (Thermo Fisher Scientific, Germany) coupled with an Orbitrap Q Exactive™ HF mass spectrometer (Thermo Fisher Scientific, Germany). The samples were injected into a Hypersil Gold column (100 mm × 2.1 mm, 1.9 μm, Thermo Fisher Scientific, United States). Separation was achieved using a 12-min linear gradient at 0.2 mL/min with eluent A (0.1% formic acid in water) and eluent B (methanol). The gradient was set as follows: 2% B, 1.5 min; 2%–85% B, 3 min; 85%–100% B, 10 min; 100%–2% B, 10.1 min; and 2% B, 12 min. The Q Exactive™ HF mass spectrometer was operated in positive/negative polarity mode with a spray voltage of 3.5 kV, capillary temperature of 320 ℃, sheath gas flow rate of 35 psi, auxiliary gas flow rate of 10 L/min, S-lens RF level 60, and auxiliary gas heater temperature of 350 ℃. Detection and analysis were performed by Shanghai Biotree Biomedical Technology Co., Ltd., Shanghai, China.

#### Statistical analysis

Raw UPLC-MS/MS data were processed using Compound Discoverer 3.3 (Thermo Fisher Scientific) for peak identification, peak alignment (5 ppm mass tolerance), and relative quantification. Compound identification was performed by matching MS/MS spectra to the mzCloud database (mass error < 5 ppm), with identities further verified using the mzVault reference library.

Data preprocessing included calculation of relative abundance and coefficient of variation (CV). Compounds with CV > 30% in the relative peak areas of QC samples were excluded. Following data filtering, compound identification and relative quantification results were finalized.

### Network pharmacology analysis

#### Active ingredients analysis of FH03FS

Active ingredient analysis of FH03FS was performed using a multi-step screening workflow. Compounds detected by UPLC-MS/MS with a relative abundance > 0.1% were initially selected. Subsequent filtering was conducted via the Traditional Chinese Medicine Systems Pharmacology (TCMSP) database based on two pharmacokinetic criteria: oral bioavailability (OB) > 20% and drug-likeness (DL) > 0.18. These thresholds were employed to achieve a balance between inclusivity and drug-like potential during the screening of natural products^19–21^.

#### ADMET properties prediction

The ADMET (Absorption, Distribution, Metabolism, Excretion, and Toxicity) properties of the identified active ingredients were predicted in silico to comprehensively evaluate their pharmacokinetic and safety profiles. This analysis utilized two complementary platforms. Parameters related to absorption, distribution, and metabolism—including gastrointestinal (GI) absorption, blood-brain barrier (BBB) permeability, P-glycoprotein (P-gp) substrate potential, inhibition of major cytochrome P450 enzymes (CYP1A2, CYP2C9, CYP2C19, CYP2D6, CYP3A4), and the bioavailability score—were obtained from the SwissADME server^22^ (http://www.swissadme.ch/). Meanwhile, key pharmacokinetic numerical parameters (volume of distribution (VD), clearance (CL), half-life (t₁/₂), and fraction unbound (Fu)) and critical toxicity endpoints (hERG inhibition, AMES mutagenicity, carcinogenicity, and human hepatotoxicity) were derived from the ADMETlab 3.0 platform^23^ (https://admetmesh.scbdd.com/).

#### Target prediction and selection

The workflow for target identification involved four key steps. First, potential protein targets of the active ingredients were predicted through SwissTargetPrediction^24^ (probability > 0; http://www.swisstargetprediction.ch/) and BATMAN-TCM^25^ (confidence score ≥ 0.84, druggable score ≥ 0.1; http://bionet.ncpsb.org.cn/batman-tcm/), with the search restricted to “*Homo sapiens*”. Concurrently, disease-associated targets for obesity and T2D were collected from the GeneCards^26^ (relevance score > 3; https://www.genecards.org/), OMIM^27^ (https://omim.org/), DrugBank^28^ (https://go.drugbank.com/), and TTD^29^ (https://db.idrblab.net/ttd/) databases. All retrieved target names were then standardized to official gene symbols using the UniProtKB database^30^ (https://www.uniprot.org/). Finally, following the removal of duplicate entries, the overlapping targets common to the FH03FS components, obesity, and T2D were identified and defined as the core target set for subsequent analysis using an online bioinformatics platform^31^ (http://www.bioinformatics.com.cn/).

#### Protein–protein interaction (PPI) network

The overlapping targets were analyzed using the STRING database^32^ (version 12.0; https://string-db.org/) with organism limited to “*Homo sapiens*”, and medium-confidence interaction score threshold ≥ 0.4. The resulting PPI network was visualized and topologically analyzed in Cytoscape (version 3.10.2). Edge weights were normalized to STRING interaction scores, and isolated nodes (degree = 0) were removed to refine network connectivity^33^.

#### Compound-target-disease network construction

A compound-target-disease network was constructed to integrate the relationships among the FH03FS active ingredients, their predicted targets, and the genes associated with obesity and T2D. This tripartite network was built and visualized using Cytoscape software, employing a node-edge topology to map the interconnections.

#### Core targets screening for anti-obesity and T2D

The CytoNCA plugin in Cytoscape was used to calculate network centrality metrics, including betweenness centrality (BC), closeness centrality (CC), degree centrality (DC), eigenvector centrality (EC), local average connectivity (LAC), and network centrality (NC), for each target^34^. Targets with metric scores exceeding the median value for all parameters were selected through intersection analysis to derive preliminary essential targets. This iterative process was repeated to finalize core targets of FH03FS active ingredients specifically associated with anti-obesity and T2D therapeutic effects.

#### Functional enrichment analysis of GO and KEGG pathways

Functional enrichment analysis of Gene Ontology (GO) and Kyoto Encyclopedia of Genes and Genomes (KEGG) pathways was conducted using the DAVID database^35^ (version 6.8; https://david.ncifcrf.gov/). The KEGG pathway database was used for functional annotation^36–38^. Analysis focused on intersection targets linked to FH03FS active ingredients with dual associations to obesity and T2D. Gene identifiers were filtered by the “official gene symbol” for Homo sapiens. For GO analysis, terms with *p* < 0.05 were considered statistically significant, whereas KEGG analysis selected pathways with false discovery rate (FDR) < 0.01. The top 10 ranked terms in biological process (BP), cellular component (CC), molecular function (MF), and key KEGG pathways were visualized via an online bioinformatics platform^39^.

### Molecular docking validation of bioactive compound-target interactions for obesity and T2D

To validate the potential interactions between bioactive compounds and therapeutic targets for obesity and T2D, molecular docking was performed on the active ingredients and the top 10 core targets. The three-dimensional structures of the active ingredients were obtained from PubChem database^34^ (https://pubchem.ncbi.nlm.nih.gov/) in MOL2 format and subsequently converted to PDB format using PyMOL 2.5.7 (Portland, OR, USA). The crystal structures of the target proteins were retrieved from the RCSB Protein Data Bank^40^(https://www.rcsb.org/).

Prior to docking, all protein structures were preprocessed to prepare for ligand binding. This involved the removal of water molecules and co-crystallized ligands, followed by the addition of hydrogen atoms. The prepared structures were then energy-minimized and converted into the PDBQT format using AutoDock Tools 1.5.6^34^. Molecular docking was conducted using AutoDock Vina 1.1.2. The resulting protein-ligand complexes were visualized in PyMOL, and binding affinities expressed in kcal/mol were calculated to quantitatively assess interaction strengths.

### Molecular dynamic simulation

Two complexes with the lowest molecular docking energies were selected for MD simulation using Gromacs 2023.2 and largely followed the standard workflow of the official GROMACS tutorial (http://www.mdtutorials.com/gmx/complex/index.html). Throughout the simulation, the system was maintained at 300 K and 1 bar. The Amber99sb-ildn force field was applied to the protein, and the GAFF force field was used for the small molecules. The system was solvated in a cubic box of TIP3P water with dimensions of 6 nm × 6 nm × 6 nm using the SPCE model, and ions were added to neutralize the system charge. The MD simulation protocol was as follows: energy minimization was first performed using the steepest descent algorithm (maximum 10,000 steps), followed by the conjugate gradient method (maximum 10,000 steps). Subsequently, the system was equilibrated under the NVT (constant volume and temperature) ensemble (100,000 steps, 2 fs time step) and then under the NPT (constant pressure and temperature) ensemble (100,000 steps, 2 fs time step). Finally, a production MD run was conducted for 100 ns for each complex^41^. The resulting trajectories were analyzed for the root mean square deviation (RMSD), root mean square fluctuation (RMSF), radius of gyration (Rg), solvent accessible surface area (SASA), the number of intermolecular hydrogen bonds, the binding free energy distribution, and the change in the distance between key amino acids and key atoms in ligands.

## Results

### UPLC-MS/MS-based phytochemical profiling

**Fig. 2:**
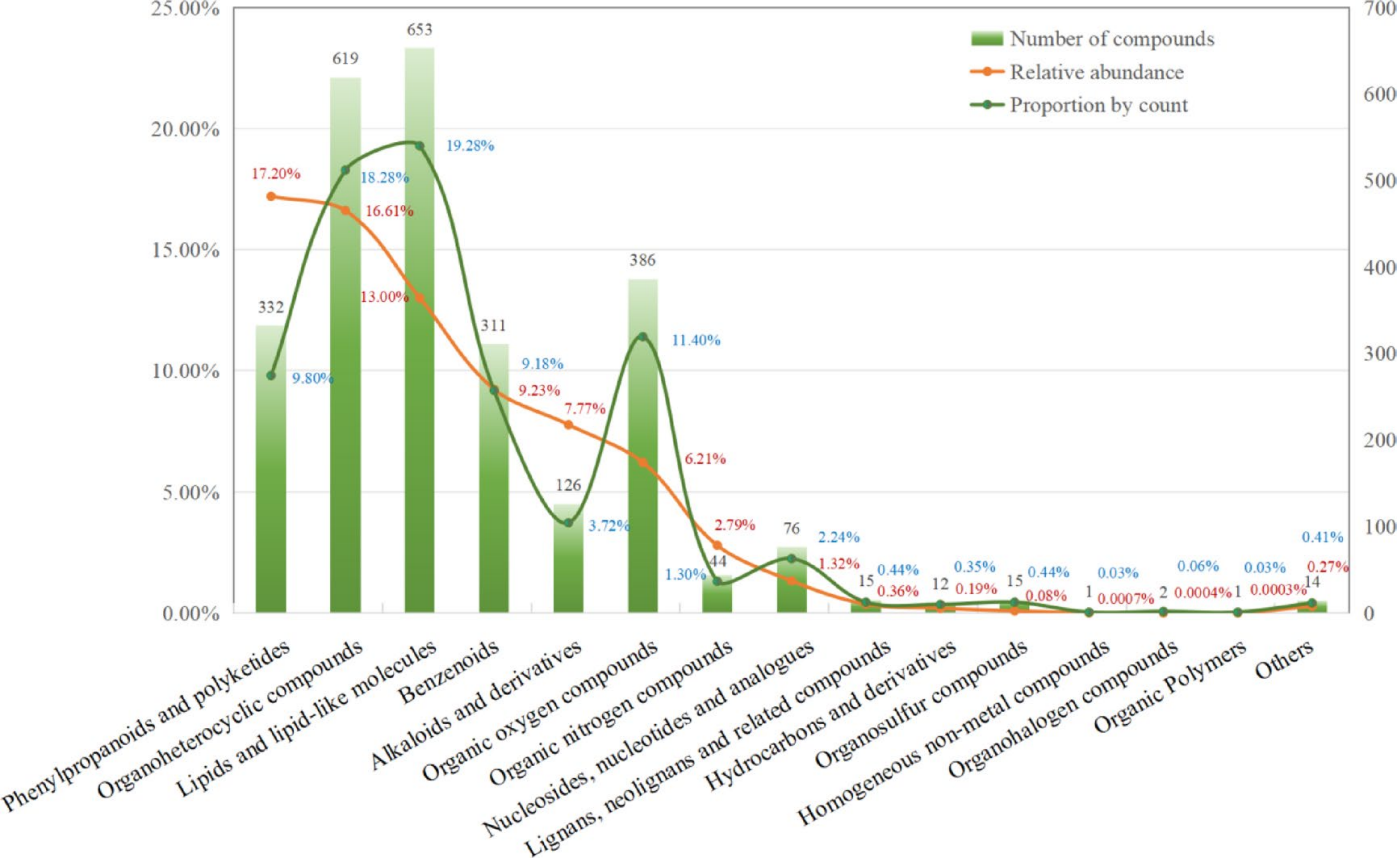
Chemical classification of compounds identified in FH03FS. The x-axis denotes chemical classes (I) categorized by the ClassyFire superclass classification system. Bar heights correspond to compound counts per class, while the orange curve illustrates relative abundance (%) and the dark green curve illustrates the percentage distribution of compounds across chemical classes.

The phytochemical composition of FH03FS was characterized using UPLC-MS/MS in both positive and negative ionization modes (see Supplementary Fig. S1 online). A total of 3,387 compounds were identified, with 2,010 detected in positive mode and 1,377 in negative mode. Compounds were categorized into 15 major classes based on the ClassyFire superclass classification system (Fig. 2). Key chemical classes included phenylpropanoids and polyketides (17.20%, relative abundance), organoheterocyclic compounds (16.61%), lipids and lipid-like molecules (13.00%), benzenoids (9.23%), alkaloids and derivatives (7.77%), and organic oxygen compounds (6.21%). Quantitative analysis further revealed six dominant classes accounting for 70.02% of total relative abundance and 71.66% of detected compounds.

### Identification of active ingredients in FH03FS​

**Fig. 3:**
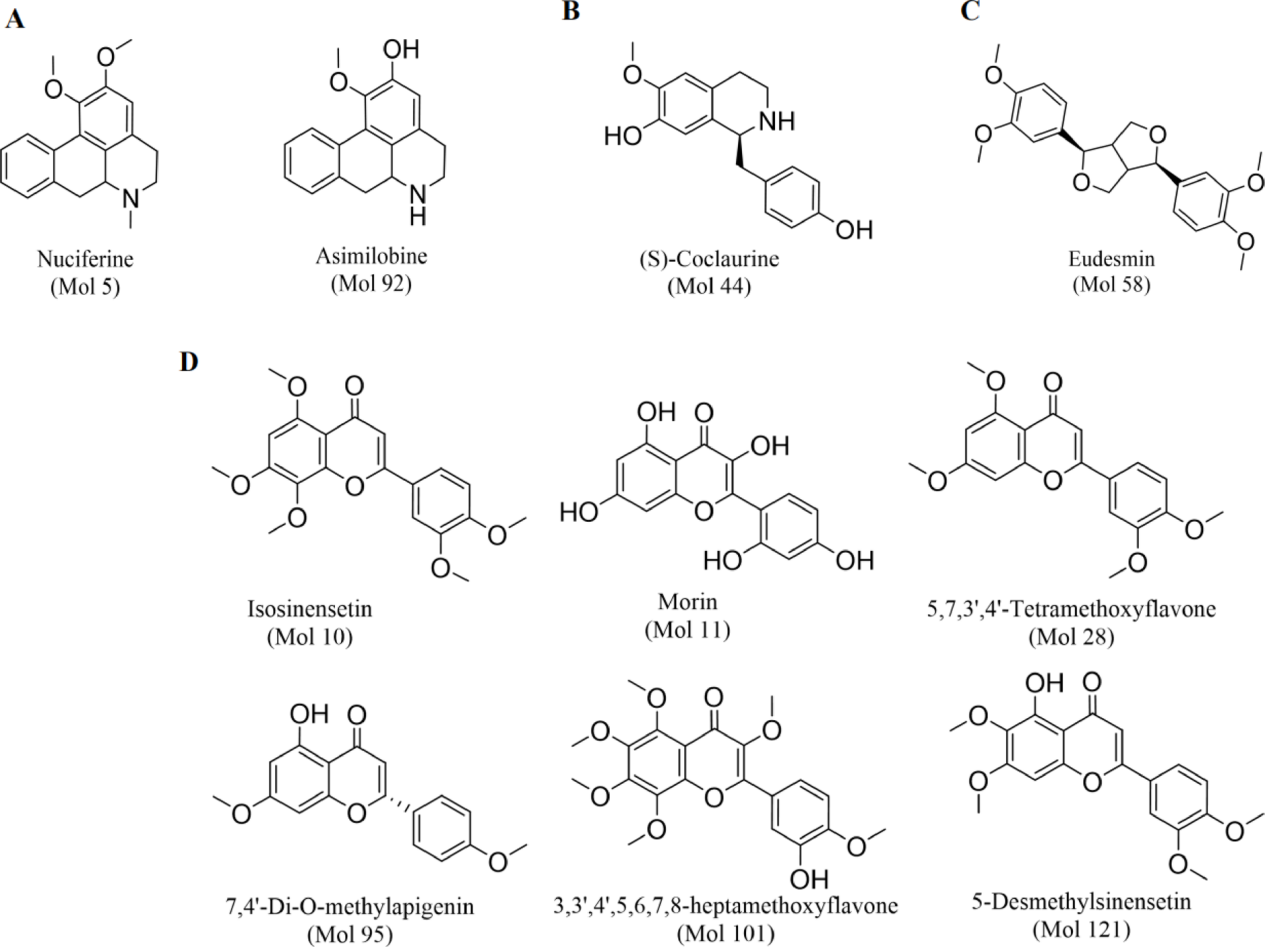
Structure of active compounds in FH03FS. (**A**) Aporphines (nuciferine and asimilobine); (**B**) Isoquinoline alkaloids ((S)-coclaurine); (**C**) Furanoid lignans (eudesmin); (**D**) Flavonoids (morin, and methoxylated derivatives).

UPLC-MS/MS analysis identified 131 compounds with relative abundance > 0.1% (see Supplementary Table S1 online). These compounds were systematically numbered from Mol 1 to Mol 131 (where “Mol” denotes molecular identifier) based on descending relative abundance. Screening via the TCMSP database identified ten compounds meeting pharmacokinetic criteria (OB > 20% and DL > 0.18) (Table 1), which were consequently designated as the active ingredients of FH03FS. The substructure-level ClassyFire categorization (Class II) systematically grouped the ten active compounds into four structural classes: aporphines represented by nuciferine (Mol 5, 3.06% relative abundance) and asimilobine (Mol 92, 0.15%); flavonoids including isosinensetin (Mol 10, 1.83%), morin (Mol 11, 1.74%), 5,7,3’,4’-tetramethoxyflavone (Mol 28, 0.55%), 7,4’-di-O-methylapigenin (Mol 95, 0.15%), and 3,3’,4’,5,6,7,8-heptamethoxyflavone (Mol 101, 0.14%), along with 5-desmethylsinensetin (Mol 121, 0.11%); isoquinolines and derivatives exemplified by (S)-coclaurine (Mol 44, 0.33%); furanoid lignans containing eudesmin (Mol 58, 0.26%). Collectively, these ten compounds accounted for 8.32% of the total relative abundance, suggesting their quantitative significance in FH03FS’s potential pharmacological activity. Structural representations are provided in Fig. 3.

**Table 1:** Pharmacokinetically screened active compounds in FH03FS.

Compound ID	Name	Relative Abundance	OB	DL	Class I	Class II	PubChem CID
Mol 5	Nuciferine	3.06%	34.43%	0.40	alkaloids and derivatives	aporphines	10146
Mol 10	Isosinensetin	1.83%	51.15%	0.44	phenylpropanoids and polyketides	flavonoids	632135
Mol 11	Morin	1.74%	46.23%	0.27	phenylpropanoids and polyketides	flavonoids	5281670
Mol 28	5,7,3’,4’-Tetramethoxyflavone	0.55%	43.68%	0.37	phenylpropanoids and polyketides	flavonoids	631170
Mol 44	(S)-Coclaurine	0.33%	42.35%	0.24	organoheterocyclic compounds	isoquinolines and derivatives	160487
Mol 58	Eudesmin	0.26%	33.29%	0.62	lignans, neolignans and related compounds	furanoid lignans	325601
Mol 92	Asimilobine	0.15%	25.34%	0.33	alkaloids and derivatives	aporphines	160875
Mol 95	7,4’-Di-O-methylapigenin	0.15%	51.54%	0.27	phenylpropanoids and polyketides	flavonoids	14057197
Mol 101	3,3’,4’,5,6,7,8-heptamethoxyflavone	0.14%	23.91%	0.58	phenylpropanoids and polyketides	flavonoids	150893
Mol 121	5-Desmethylsinensetin	0.11%	26.31%	0.41	phenylpropanoids and polyketides	flavonoids	152430

### ADMET profiling of active Ingredients​

Predicted ADMET properties for the ten active ingredients indicated favorable pharmacokinetic and safety profiles (Table 2). All compounds showed high GI absorption and a consensus Bioavailability Score of 0.55, supporting strong potential for oral bioavailability. Regarding distribution, most compounds (7/10) were predicted to cross the blood-brain barrier, while key flavonoids like morin were non-permeants, potentially reducing Central Nervous System (CNS) side effect risks. Several compounds, including nuciferine, (S)-coclaurine and asimilobine, were identified as P-glycoprotein substrates, which may affect their tissue distribution. Metabolically, significant inhibition of CYP3A4 (8/10) and CYP2D6 (8/10) was predicted, suggesting potential for drug-drug interactions^42^. Toxicity predictions were largely favorable: most compounds showed low hERG inhibition risk and were non-mutagenic in AMES testing, and hepatotoxicity risks were generally low.

**Table 2:** Predicted ADMET properties of the active ingredients in FH03FS.

Compound ID	Mol 5	Mol 10	Mol 11	Mol 28	Mol 44	Mol 58	Mol 92	Mol 95	Mol 101	Mol 121
MW (g/mol)	295.4	372.4	302.2	342.3	285.3	386.4	267.3	298.3	432.4	358.3
TPSA (Å²)	21.7	76.36	131.36	67.13	61.72	55.38	41.49	68.9	94.82	87.36
H-bond Donor	0	0	5	0	3	0	2	1	0	1
H-bond Acceptor	3	7	7	6	4	6	3	5	9	7
GI Absorption	High	High	High	High	High	High	High	High	High	High
BBB Permeant	Yes	Yes	No	Yes	Yes	Yes	Yes	Yes	No	No
P-gp Substrate	Yes	No	No	No	Yes	No	Yes	No	No	No
CYP1A2 Inhibitor	No	No	Yes	Yes	No	No	Yes	Yes	No	Yes
CYP2C9 Inhibitor	No	Yes	No	Yes	No	No	No	Yes	Yes	Yes
CYP2C19 Inhibitor	No	No	No	Yes	No	No	No	Yes	No	No
CYP2D6 Inhibitor	Yes	No	Yes	Yes	Yes	Yes	Yes	Yes	No	Yes
CYP3A4 Inhibitor	Yes	Yes	Yes	Yes	No	Yes	Yes	Yes	No	Yes
Bioavailability Score	0.55	0.55	0.55	0.55	0.55	0.55	0.55	0.55	0.55	0.55
VD (L/kg)	0.46	–0.13	–0.65	–0.07	–0.26	0.01	0.32	–0.21	–0.09	–0.38
CL (mL/min/kg)	9.92	7.2	2.98	6.59	6.6	7.43	5.26	6.01	6.94	5.65
t₁/₂ (h)	3.99	1.9	1.93	1.41	1.76	2.61	1.54	0.68	1.61	1.51
Fu (%)	19.39	5.15	3.04	8.08	49.29	15.47	48.18	5.35	5.98	2.31
hERG Inhibitor-10 μm (Risk)	0.71	0.51	0.47	0.46	0.74	0.56	0.65	0.50	0.55	0.46
Ames Test	0.69	0.47	0.57	0.62	0.53	0.65	0.83	0.72	0.27	0.50
Carcinogenicity	0.78	0.82	0.66	0.84	0.32	0.53	0.30	0.85	0.82	0.75
Human Hepatotoxicity	0.46	0.45	0.37	0.45	0.54	0.59	0.68	0.43	0.45	0.43

Collectively, this ADMET profiling suggests that the predominant bioactive ingredients in FH03FS possess promising pharmacokinetic properties and an acceptable safety profile, supporting their further investigation as functional food components for metabolic health.

### Active ingredients and their potential targets against obesity and T2D

**Fig. 4:**
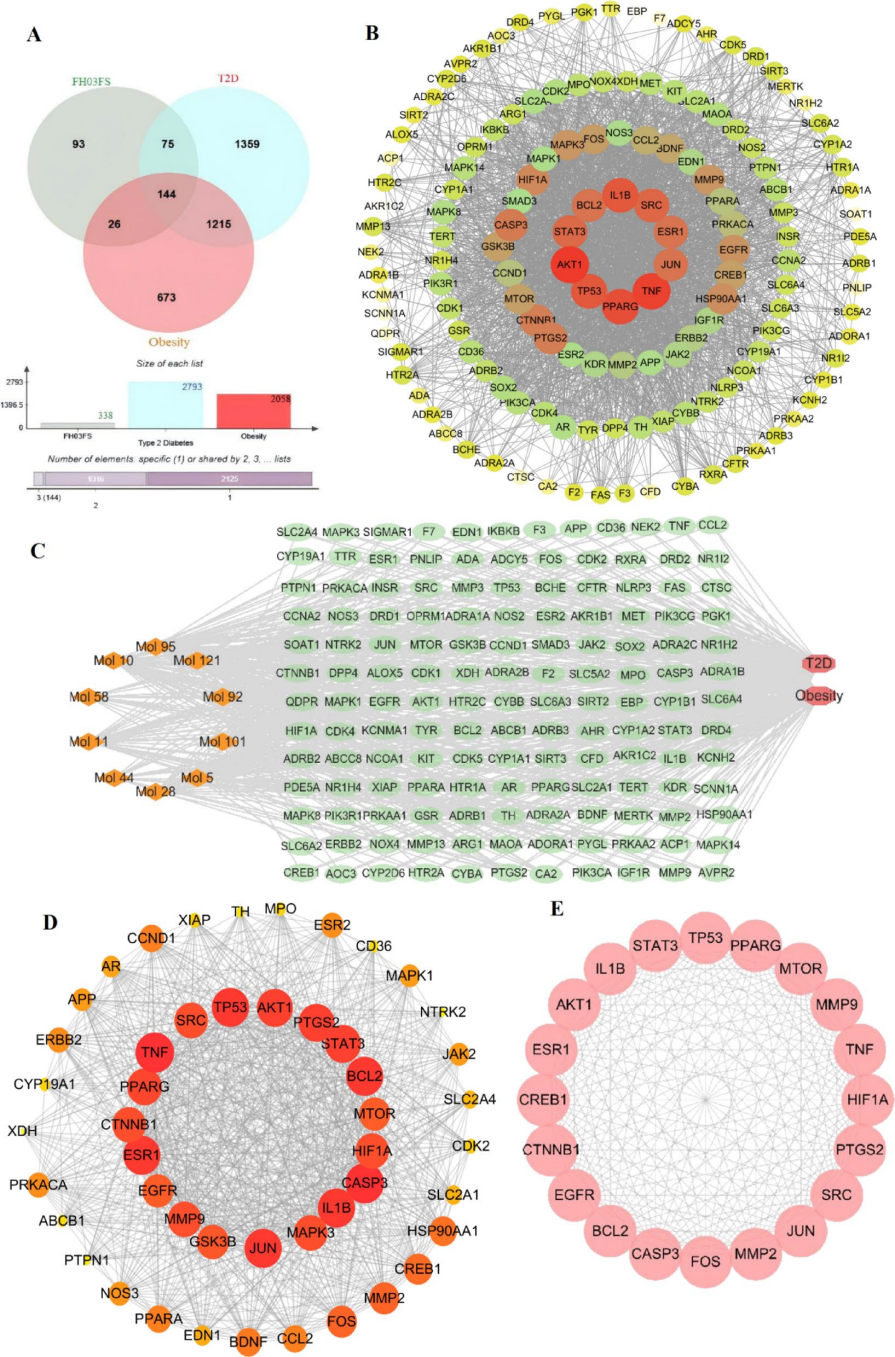
Network pharmacology analysis of FH03FS active ingredients against obesity and T2D: (**A**) Venn diagram showing overlapping targets of FH03FS (green) with obesity-associated (red) and T2D-associated (blue) genes. Intersections indicate shared therapeutic targets. (**B**) Protein–protein interaction (PPI) network of the overlapping targets identified in (**A**). Node size reflects degree centrality; yellow-to-red gradient indicates association strength (red: strongest, yellow: weakest). (**C**) Compound-target-disease network. Diamond nodes represent FH03FS active ingredients, the green ellipses denote the targets, and octagons indicate obesity and T2D diseases. Edges depict interaction relationships. (**D**) Top 48 high-value targets screened from the PPI network using six topological parameters (BC: betweenness centrality, CC: closeness centrality, DC: degree centrality, EC: eigenvector centrality, LAC: local average connectivity, NC: network centrality). Threshold: median score of 144 initial genes. (**E**) 20 core targets further filtered from (**D**) by applying stricter topological criteria (all six parameters above median).

Pharmacological network analysis identified 338 potential protein targets for the ten active ingredients via BATMAN-TCM and SwissTargetPrediction databases. Concurrently, disease-associated targets were retrieved for obesity (2058 targets) and T2D (2793 targets) from GeneCards, OMIM, DrugBank, and TTD databases. Bioinformatics analysis identified 144 overlapping targets between the compound-target network and disease-associated proteins (Fig. 4A), suggesting potential shared molecular mechanisms underlying FH03FS’s potential dual therapeutic effects against obesity and T2D comorbidity.

### PPI network construction and compound-target-disease analysis

The 144 overlapping targets were analyzed using the STRING database (version 12.0) to construct a PPI network. After removing disconnected nodes, the final network comprised 144 nodes (targets) and 2464 edges (interactions), with an average clustering coefficient of 0.610 and functional enrichment significance of *p* < 1.0 × 10⁻¹⁶. Topological analysis in Cytoscape (v3.10.2) visualized node size and color intensity proportional to degree centrality (range: 1–97; median: 28) (Fig. 4B). The constructed compound-target-disease network integrated the active ingredients of FH03FS, 144 predicted targets (represented as ellipse nodes), and disease entities (represented as octagonal nodes, including obesity and T2D) (Fig. 4C), with edges representing compound-target binding and target-disease associations, providing a systems-level view of the potential therapeutic mechanisms.

### Identification of core therapeutic targets for obesity and T2D intervention

Using the CytoNCA plugin (v2.1.6) in Cytoscape (v3.10.2), we systematically screened the PPI network to prioritize targets based on six topological parameters: BC, CC, DC, EC, LAC, NC. From the 144 overlapping genes associated with FH03FS active ingredients, obesity, and T2D, initial screening selected targets scoring above the median for all parameters, yielding 48 candidate genes (Fig. 4D; Supplementary Tables S2, S3). Further refinement identified 20 core targets demonstrating the strongest network influence: JUN, TNF, PPARG, TP53, MMP2, AKT1, PTGS2, STAT3, BCL2, MTOR, IL1B, CASP3, HIF1A, FOS, CTNNB1, SRC, EGFR, CREB1, MMP9, and ESR1 (Fig. 4E; Supplementary Table S4).

### Gene ontology (GO) and KEGG pathway enrichment analysis​​

**Fig. 5:**
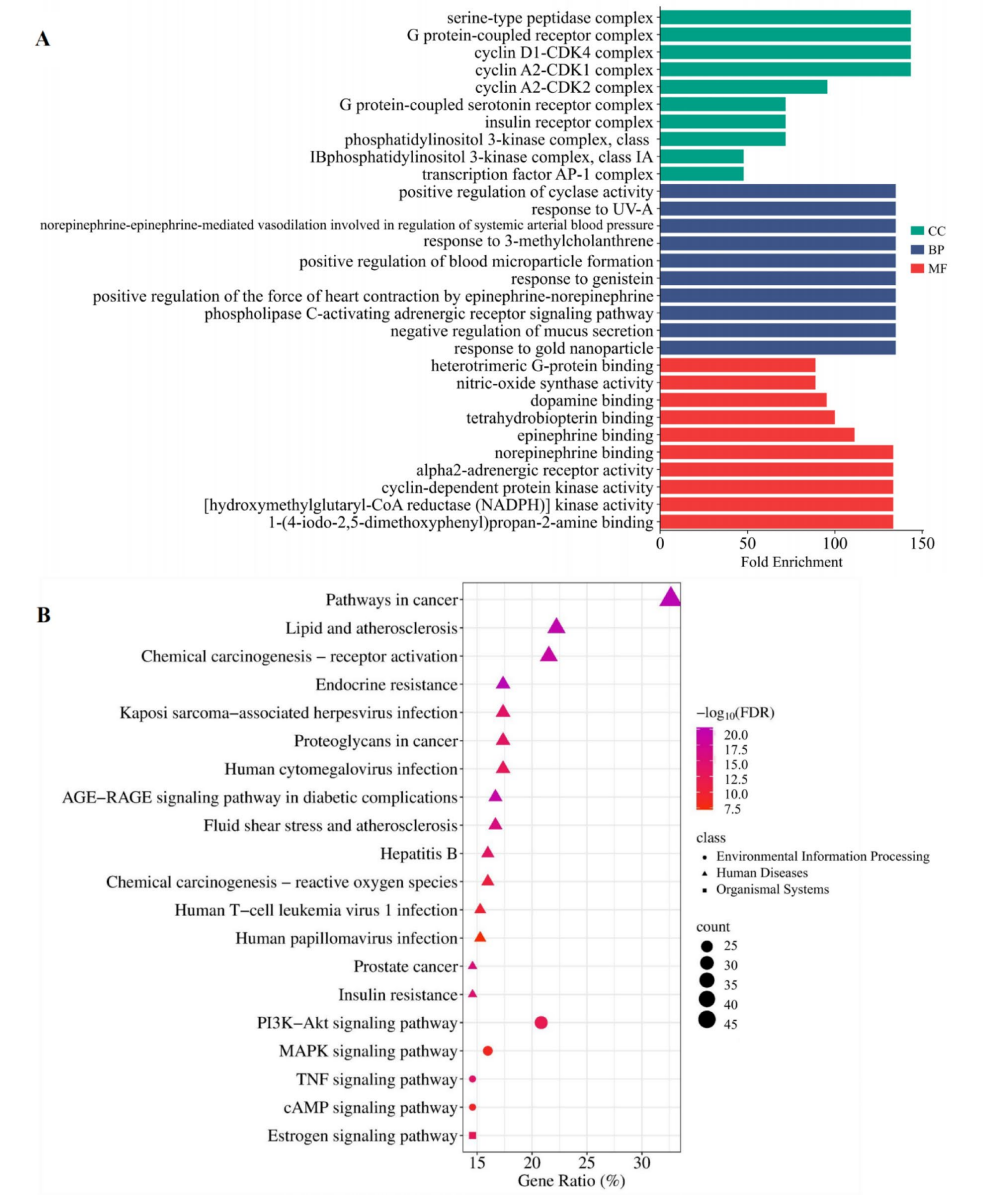
Functional enrichment analysis of FH03FS active ingredients against obesity and T2D. (**A**) Gene Ontology (GO) enrichment analysis illustrates the top 10 significantly enriched terms (*p* < 0.05) across three categories: cellular components (green bars), biological processes (blue bars), and molecular functions (red bars). The x-axis denotes fold enrichment values, with bar length proportional to enrichment significance. (**B**) Kyoto Encyclopedia of Genes and Genomes (KEGG) pathway enrichment analysis displays the top 20 significantly enriched pathways (FDR < 0.01), categorized into three systems: human diseases (triangles), environmental information processing (circles), and organismal systems (squares). The x-axis indicates gene ratio (%), while the color gradient (red to dark purple) represents decreasing FDR values.

Comprehensive GO and KEGG pathway enrichment analyses were performed to elucidate the multi-target mechanisms of the ten active ingredients in FH03FS against obesity and T2D. GO analysis identified 1072 statistically significant terms (*p* < 0.05), including 97 CC, 755 BP, and 220 MF. Key enriched CC terms included serine-type peptidase complex, G protein-coupled receptor complex, and cyclin D1-CDK4 complex. Top BP terms highlighted regulatory functions such as positive regulation of cyclase activity, response to UV-A, and norepinephrine-mediated vasodilation. MF analysis revealed significant enrichment in norepinephrine binding, alpha2-adrenergic receptor activity, and cyclin-dependent protein kinase activity (Fig. 5A).

KEGG pathway^36–38^analysis identified 173 significantly enriched pathways (FDR < 0.01), predominantly associated with three biological systems (Fig. 5B):

Human diseases: Pathways in cancer (32.6% gene ratio), lipid and atherosclerosis (22.2%), AGE-RAGE signaling in diabetic complications (16.7%), and insulin resistance (14.6%);

Environmental information processing: PI3K-Akt signaling (20.8% gene ratio), MAPK signaling (16.0%), cAMP signaling (14.6%), and TNF signaling (14.6%).

Organismal systems: Estrogen signaling pathway (14.6% gene ratio).

These findings collectively demonstrate that FH03FS exerts potential anti-obesity and anti-T2D effects through coordinated regulation of interconnected pathways spanning metabolic regulation, inflammatory response, and cellular signaling.

### Molecular Docking analysis

**Fig. 6:**
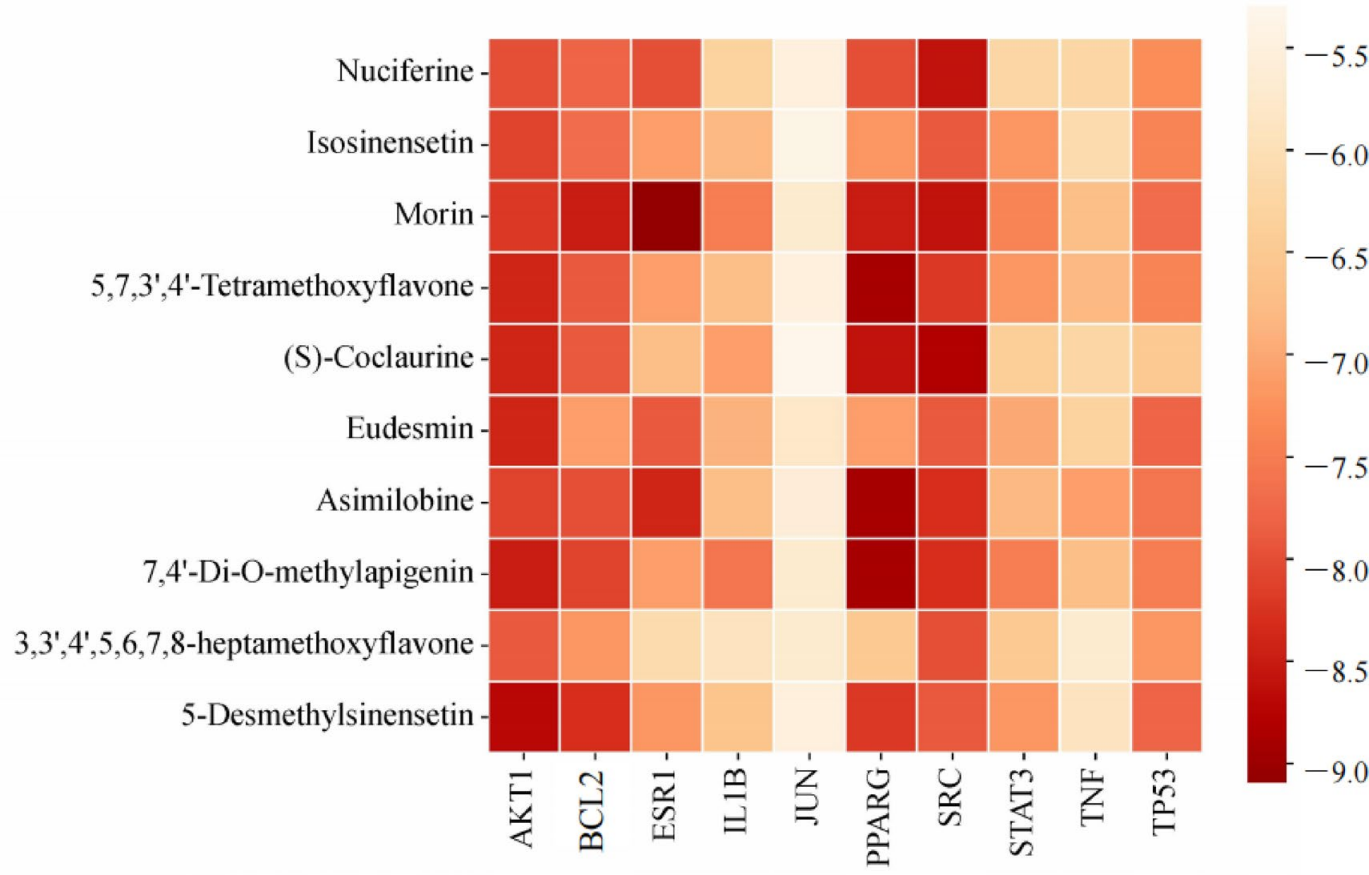
Heatmap visualization of molecular docking binding energies between FH03FS bioactive compounds and core therapeutic targets. The color gradient (orange to red) represents binding affinities from weakest (orange, − 5.3 kcal/mol) to strongest (red, − 9.1 kcal/mol).

**Fig. 7:**
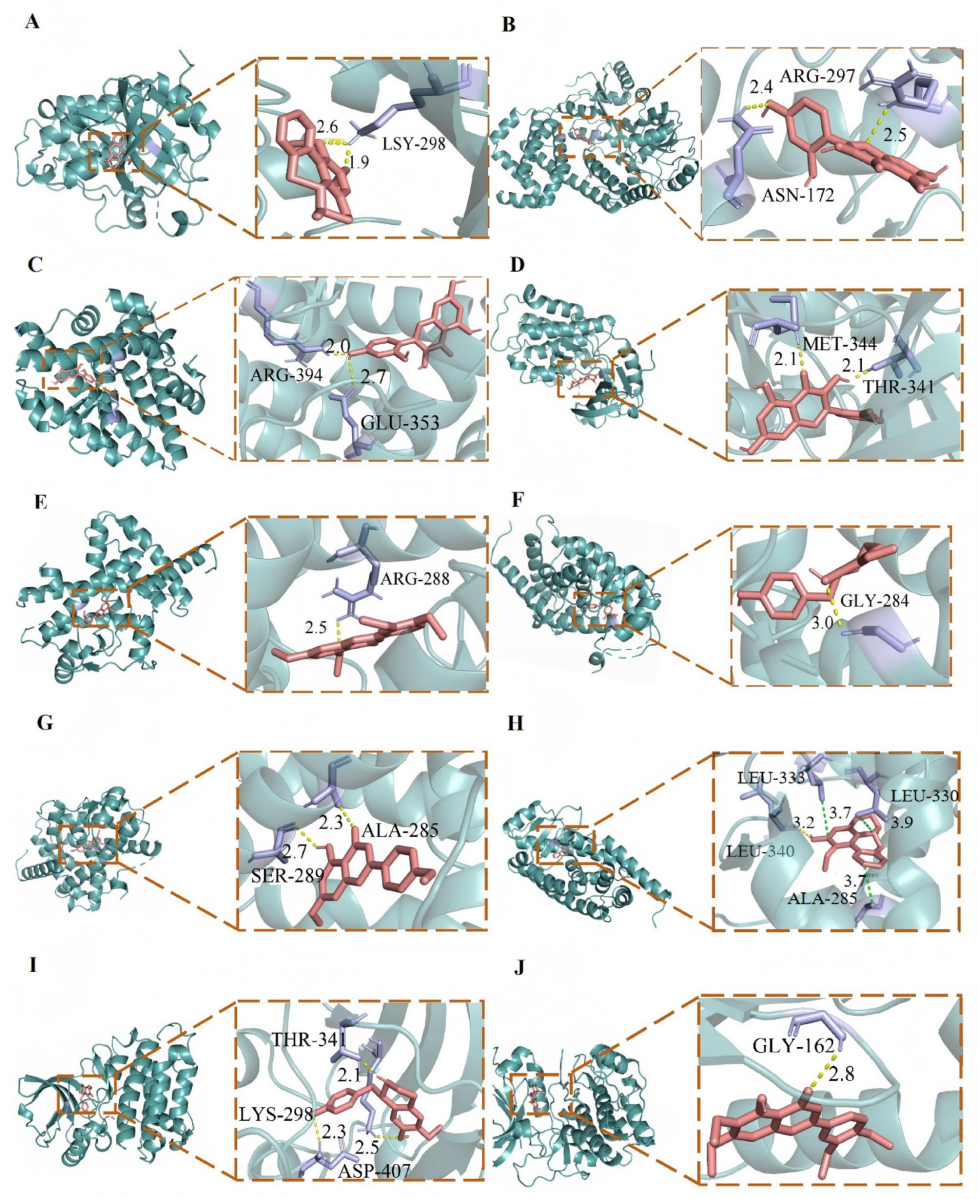
Molecular docking visualization of high-affinity compound-target interactions. Red sticks represent FH03FS active ingredients, with hydrogen bonds shown as yellow dashed lines: (**A**) Nuciferine-SRC; (**B**) Morin-BCL2; (**C**) Morin-ESR1; (**D**) Morin-SRC; (**E**) 5,7,3’,4’-Tetramethoxyflavone-PPARG; (**F**) (S)-coclaurine-PPARG; (**G**) 7,4’-Di-O-methylapigenin-PPARG; (**H**) Asimilobine-PPARG; (**I**) (S)-coclaurine-SRC; (**J**) 5-Desmethylsinensetin-AKT1.

Molecular docking was performed to investigate the interactions between ten ingredients from FH03FS and ten core targets such as AKT1, TNF, PPARG, TP53, IL1B, SRC, STAT3, JUN, BCL2, and ESR1, which were selected as the highest-degree nodes from the 20 core genes identified through CytoNCA-based topological analysis (Supplementary Tables S2, S4), representing the most connected hubs in the PPI network. The results demonstrated strong binding affinities for all tested pairs (Fig. 6). Notably, several key targets were engaged by multiple compounds with high affinity. For instance, the peroxisome proliferator-activated receptor gamma (PPARG) showed strong binding (from − 8.9 to − 8.6 kcal/mol) to several compounds, including 5,7,3’,4’-tetramethoxyflavone, (S)-coclaurine, asimilobine, and 7,4’-di-O-methylapigenin. Conversely, some compounds, such as the flavonoid morin, exhibited a broad targeting profile, with exceptional binding affinity to ESR1 (− 9.1 kcal/mol), BCL2 (− 8.5 kcal/mol), and SRC (− 8.6 kcal/mol). Structural analysis revealed that aporphines and flavonoids were the most active compound classes, forming key hydrogen bonds, suggesting their crucial role in FH03FS’s anti-obesity and anti-T2D effects. All high-affinity pairs specifically mentioned above, including Nuciferine-SRC, Morin-ESR1/BCL2/SRC, PPARG-binding compounds, (S)-coclaurine-SRC, and 5-Desmethylsinensetin-AKT1 were visualized in PyMOL (Fig. 7).

This multi-target engagement profile provides a structural basis for FH03FS’s synergistic pharmacological effects through key obesity/T2D-related signaling axes.

### Molecular dynamic simulation analysis

The structural stability and binding affinity of the Morin-ESR1 and Asimilobine-PPARG complexes were assessed through 100-ns MD simulations. Both systems demonstrated excellent stability throughout the simulation period.

**Fig. 8:**
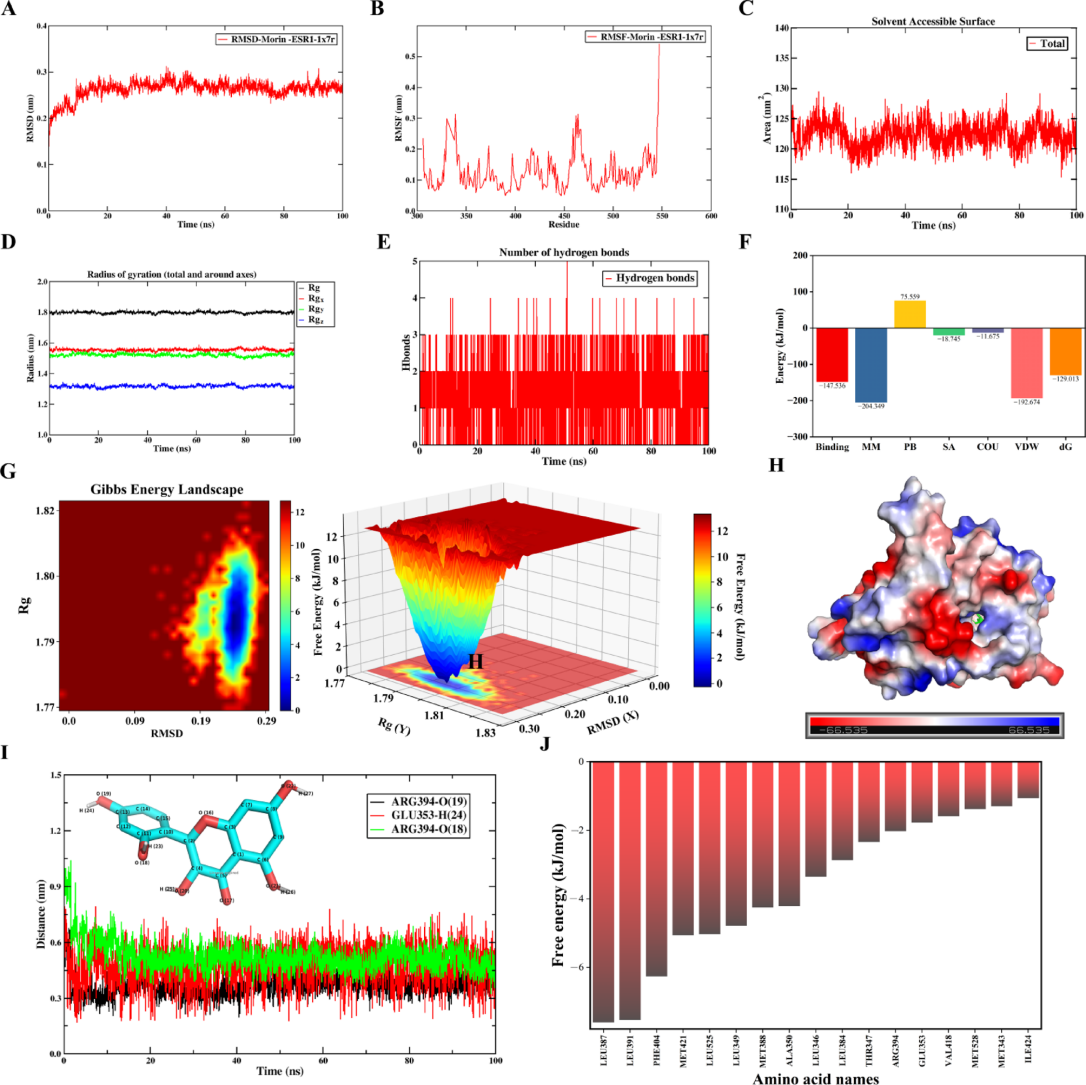
Molecular dynamics (MD) simulation of the Morin-ESR1 complex over 100 ns. (**A**) Root mean square deviation (RMSD) curve of the complex. (**B**) Root mean square fluctuation (RMSF) curve of ESR1. (**C**) Solvent accessible surface area (SASA) curve of ESR1. **(D**) Radius of gyration (Rg) curve of the complex. (**E**) Number of intermolecular hydrogen bonds of the complex. (**F**) The Molecular Mechanics/Poisson-Boltzmann Surface Area (MM-PBSA) binding free energy decomposition analysis. The bar graph illustrates the contribution of individual energy components to the total binding free energy, including molecular mechanics (MM), polar solvation (PB), non-polar solvation (SA), electrostatic (COU), and van der Waals (VDW) terms. “Binding” represents the Gibbs free energy change of binding, which is the sum of the Gibbs free energy changes from MM, PB, and SA; “dG” denotes the calculated binding energy after considering the entropy change (dS). (**G**) Free energy landscape (FEL) of the complex projected onto RMSD and Rg. (**H**) Surface electrostatic potential mapping of the complex around the binding pocket. Red indicates negatively charged regions, blue indicates positively charged regions, and white indicates neutral (uncharged) regions. (**I**) Change in the distance between key amino acids and key atoms in the morin. (**J**) Free energy decomposition of the complex.

**Fig. 9:**
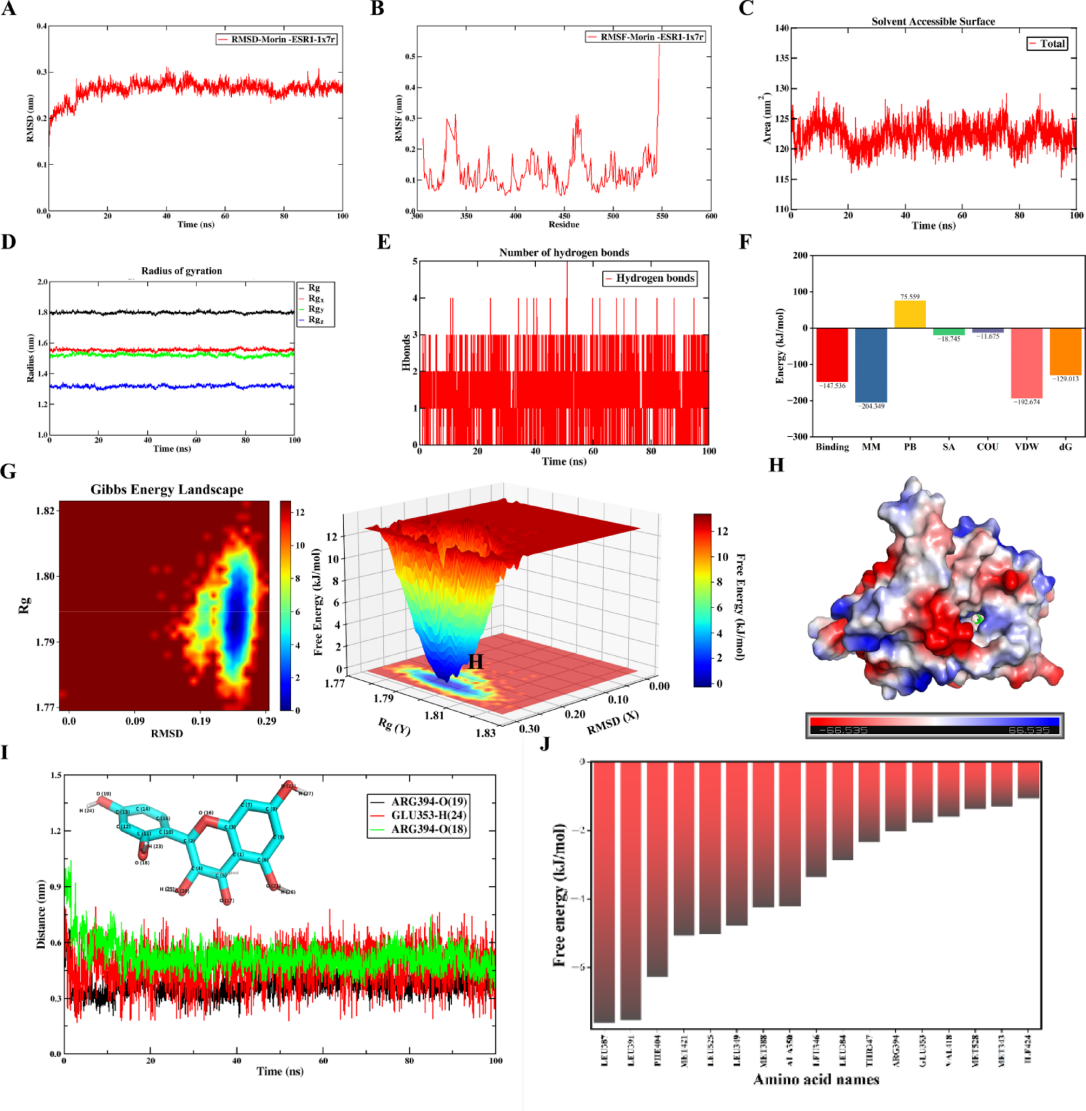
MD simulation of the Asimilobine-PPARG complex over 100 ns. (**A**) RMSD curve of the complex. (**B**) RMSF curve of PPARG. (**C**) SASA curve of PPARG. (**D**) Rg curve of the complex. (**E**) Number of intermolecular hydrogen bonds of the complex. (**F**) MM-PBSA Binding Free Energy Decomposition Analysis. (**G**) FEL of the complex projected onto RMSD and Rg. (**H**) Surface electrostatic potential mapping of the complex. (**I**) Change in the distance between key amino acids and key atoms in asimilobine. (**J**) Free energy decomposition of the complex.

The RMSD values stabilized at approximately 0.26 nm and 0.28 nm for Morin-ESR1 and Asimilobine-PPARG after the initial 20–30 ns, respectively (Figs. 8A and 9A), indicating that the complexes reached a stable equilibrium. The RMSF revealed higher flexibility in the loop regions, while the binding pocket residues exhibited remarkably low fluctuations, particularly in the Morin-ESR1 complex (Figs. 8B and 9B). The average SASA values remained consistent at 123 nm² for the Morin-ESR1 complex and 145 nm² for the Asimilobine-PPARG complex (Figs. 8C and 9C). The Rg remained stable throughout the simulation (Figs. 8D and 9D), implying a compact overall structure without significant expansion or unfolding. Hydrogen bond analysis confirmed the formation of stable intermolecular interactions, with an average of 3 and 1.5 hydrogen bonds maintained in the Morin-ESR1 and Asimilobine-PPARG complexes, respectively (Figs. 8E and 9E).

To quantitatively evaluate the binding strength, the Molecular Mechanics/Poisson-Boltzmann Surface Area (MM-PBSA) method was employed to calculate the binding free energy. The results revealed strongly negative values of ΔG for Morin-ESR1 and Asimilobine-PPARG (Figs. 8F and 9F), affirming spontaneous and high-affinity binding. Energy decomposition analysis indicated that van der Waals (VDW) interactions were the major driving force for Morin-ESR1 binding, whereas electrostatic interactions (COU) contributed more substantially to Asimilobine-PPARG stability.

The free energy landscape (FEL) constructed based on RMSD and Rg displayed a single, deep global minimum (blue region) for each system (Figs. 8G and 9G), confirming that the simulated complexes predominantly populated a stable, low-energy conformation.

The surface electrostatic potential around the binding pockets (Figs. 8H and 9H) were examined. For the Morin-ESR1 complex, the binding interface was characterized by large continuous white (neutral) surfaces, indicative of extensive hydrophobic patches, punctuated by key complementary polar interactions. In contrast, the interface of the Asimilobine-PPARG complex displayed more distinct and localized strong complementary electrostatic patches (red-blue opposition), which aligns with its greater reliance on electrostatic contributions for binding.

To gain atomic-level insight into the stability of the key interactions identified by molecular docking and MM-PBSA, we monitored the time-dependent distances between critical atoms of the ligands and their binding pocket residues (Figs. 8I and 9I). For the Morin-ESR1 complex, the distances corresponding to the pivotal hydrogen bonds with ARG394 remained stable within the canonical bonding range (approx. 0.25–0.30 nm) throughout the entire simulation (Fig. 8I). This persistent polar interaction network firmly anchors the ligand within the binding site.

Analysis of the specific atomic distances in the Asimilobine-PPARG complex (Fig. 9I) confirmed the stability of the binding mode. While the hydrogen bond with LEU340 was dynamic, the hydrophobic interactions with residues such as LEU333 remained consistently tight. Notably, the ligand exhibited a minor conformational adjustment around 80 ns, resulting in strengthened hydrophobic contacts with LEU330 and ALA285, as evidenced by a concerted decrease in their atomic distances. This local optimization, occurring within a globally stable framework, further stabilized the complex in the latter part of the simulation and is indicative of a high-affinity binding pose achieving its energy minimum.

Figures 8J and 9J present the binding free energy decomposition for key residues in the Morin-ESR1 and Asimilobine-PPARG complexes, respectively. Consistent with the total binding energies (Figs. 8F and 9F), the interaction in Morin-ESR1 is predominantly driven by VDW forces, with hydrophobic residues such as LEU387 making major contributions (Fig. 8J). In contrast, binding in the Asimilobine-PPARG complex relies primarily on COU, largely governed by acidic residues including GLU295 (Fig. 9J).

## Discussion

The escalating global syndemic of obesity and T2D is fundamentally driven by a pathophysiological interplay between metabolic dysfunction and chronic inflammation. This interplay presents a formidable challenge to conventional single-target therapies. It has spurred growing interest in MFH materials, which offer a paradigm for developing safe, multi-targeted dietary interventions rooted in traditional wisdom. Against this backdrop, the present study investigates FH03FS, a sterilized, probiotic-fermented beverage formulated from five MFH ingredients. Our integrated in silico approach demonstrates that the therapeutic potential of FH03FS likely stems from the collective action of its primary bioactive constituents—aporphines and flavonoids—which exhibit a multi-target capacity against key nodes in metabolic inflammatory pathways. By focusing squarely on the final sterilized product, this research addresses a pivotal translational gap, providing mechanistic insights directly relevant to the commercial, ready-to-consume beverage.

The potential efficacy of FH03FS is underpinned by its complex phytochemical profile, which is dominated by bioactive aporphines and flavonoids. Beyond their relative abundance, these compounds are characterized by favorable predicted ADMET properties, including high GI absorption and a consensus bioavailability score of 0.55. Such favorable ADMET profiles support their potential for oral bioavailability when used as components in functional beverages^22,23^.

Mechanistically, these constituents engage a network of targets to address core aspects of metabolic inflammation. The aporphine nuciferine, the most abundant active component, is a well-established modulator of lipid metabolism via AMP-activated protein kinase (AMPK) activation^43,44^, and it demonstrated efficacy in improving hepatic steatosis and adipose inflammation^45,46^. Its structural analog, asimilobine, complements these effects by activating PPARG^47^. Among the flavonoids, isosinensetin presents a dual anti-diabetic mechanism by stimulating GLP-1 secretion^48^ and activating the nuclear factor erythroid 2-related factor 2 (Nrf2) pathway^49^.

The flavonoid morin exhibited a particularly broad multi-target profile, potentially preserving pancreatic β-cell function by inhibiting human islet amyloid polypeptide (hIAPP) aggregation and NACHT, LRR and PYD domains-containing protein 3 (NLRP3) inflammasome activation^50,51^, while simultaneously enhancing insulin sensitivity and glucose uptake^52^. Its predicted inability to cross the blood-brain barrier is a notable safety advantage for a dietary intervention^22,23^. Other methoxylated flavonoids, such as 5,7,3’,4’-tetramethoxyflavone, further contribute to this polypharmacological profile through mechanisms like α-glucosidase inhibition^53–55^. Collectively, the multi-target bioactivities of these key phytochemicals, coupled with their promising pharmacokinetic and safety profiles, provide a compelling phytochemical foundation for the observed network pharmacology predictions and the postulated efficacy of FH03FS against obesity and T2D.

Building upon this bioactive profile, our network pharmacology analysis systematically pinpointed 20 core protein targets through which these phytochemicals may exert their concerted effects. These targets, including AKT1, TNF, PPARG, ESR1, and SRC, are not isolated entities but function as an interconnected network that regulates glucose homeostasis, lipid metabolism, and inflammatory responses. This interconnected regulatory network thereby provides a systems-level explanation for the beverage’s potential efficacy.

Several key targets emerge as central hubs within this network. RAC-alpha serine/threonine-protein kinase (AKT1) serves as a critical node, enhancing insulin sensitivity through the PI3K-Akt signaling by promoting glucose transporter type 4 (GLUT4)-mediated glucose uptake and inhibiting hepatic gluconeogenesis via forkhead box protein O1 (FOXO1)^56–58^, while also counteracting inflammation by suppressing TNF/NF-κB signaling^59^. PPARG plays a dual role, improving insulin sensitivity via PI3K-Akt activation^60,61^, while modulating vascular inflammation^62^, though its pro-adipogenic activity warrants balanced targeting^63^.

The inflammatory cascade is significantly represented by Tumor necrosis factor-alpha (TNF) and Interleukin-1 beta (IL1B), which drive insulin resistance and β-cell dysfunction^64,65^, and are functionally linked to the AGE-RAGE signaling axis^66,67^. Furthermore, Estrogen receptor (ESR1) integrates hormonal and metabolic regulation by suppressing appetite and adipogenesis^68,69^, while Signal transducer and activator of transcription 3 (STAT3) and Apoptosis regulator Bcl-2 (BCL2) offer complementary protection to pancreatic β-cell against lipotoxicity and apoptosis^70–72^. The regulatory landscape is further refined by targets exhibiting tissue-specific duality, such as Cellular tumor antigen p53 (TP53)^73,74^ and Proto-oncogene tyrosine-protein kinase Src (SRC)^75–77^, which modulate metabolic processes in a context-dependent manner.

This target ensemble aligns closely with the multi-target capabilities of FH03FS’s key constituents, suggesting that aporphines and flavonoids likely produce their systemic effects by co-modulating this core network—particularly the PI3K-Akt, PPAR, and inflammatory signaling axes—rather than through isolated target engagement.

To further delineate the functional context of these targets, GO and KEGG analyses collectively revealed a multi-layered regulatory network through which FH03FS may ameliorate obesity and T2D. The significantly enriched G protein-coupled receptor (GPCR) activity, particularly targeting adrenergic receptors ADRB1/2/3 mediating catecholamine-driven lipolysis^78,79^, suggests enhanced energy expenditure, while enrichment of insulin receptor-PI3K complexes^56,68^ supports amplified PI3K-Akt signaling for GLUT4-mediated glucose uptake and FOXO1-inhibited hepatic gluconeogenesis inhibition^56^. Transcriptional regulation emerges as another key layer, with enriched AP-1^80^ and cyclin D1-CDK4 complexes^81–83^, potentially coordinating adipocyte differentiation and β-cell proliferation. These mechanisms are complemented by serotonergic regulation via HTR2C/3A receptors^84^, indicating dual appetite suppression and glycemic control.

KEGG mapping reveals systemic modulation of core pathways: the PI3K-Akt axis^56^ restores insulin signaling, further supported by nuciferine’s AMPK-dependent lipid regulation^45,46^, and morin’s inhibition of TNF-induced IRS-1 phosphorylation^85^ and JNK/MAPK activation^86,87^. The AGE-RAGE pathway attenuation^66,67^, combined with morin’s suppression of hIAPP aggregation^50,51^, and asimilobine’s PPARG activation^47^, protects against diabetic complications. Concurrently, PPAR signaling^60,61^, alongside methoxylated flavonoids’ α-glucosidase inhibition^53^ and nuciferine’s anti-steatotic effects^45,46^, addresses dyslipidemia. These enriched pathways collectively demonstrate that FH03FS’s bioactive constituents target interconnected metabolic inflammatory processes through insulin sensitization, lipid homeostasis restoration, and inflammatory suppression.

The multi-target and multi-pathway modulatory potential of FH03FS, as predicted by network pharmacology and functional enrichment, was subsequently preliminarily validated at the molecular level through computational approaches. Molecular docking showed strong binding interactions between the key bioactive compounds and the core targets.

The aporphine nuciferine showed high-affinity binding (≤ − 8.0 kcal/mol) to multiple targets, including SRC, AKT1, ESR1, and PPARG. Its interaction with SRC suggests a potential role in limiting adipogenesis and improving insulin sensitivity^75,86^. Binding to AKT1 indicates it may enhance glucose metabolism through the PI3K-Akt signaling^57^. Nuciferine’s affinity for ESR1 and PPARG further supports a regulatory role in lipid metabolism^49,61^. Among flavonoids, morin exhibited strong binding to ESR1, BCL2, and SRC (≤ − 8.5 kcal/mol), pointing to possible anti-adipogenic^49^ and β-cell protective effects^71^. Its engagement with SRC may also facilitate indirect modulation of insulin signaling. Additionally, 5-desmethylsinensetin bound tightly to AKT1 (− 8.7 kcal/mol), reinforcing potential insulin-sensitizing activity. Other methoxylated flavonoids, including 7,4’-di-O-methylapigenin and 5,7,3’,4’-tetramethoxyflavone, also displayed high affinity for PPARG, supporting a collective role in lipid regulation. Morin’s interaction with STAT3 and TNF further suggests anti-inflammatory potential via JAK/STAT and NF-κB pathways^52^. These results structurally validate the multi-target mechanisms predicted by network pharmacology.

To further assess the stability of these predicted interactions, we performed 100-ns MD simulations on the two complexes with the lowest binding energies: Morin-ESR1 and Asimilobine-PPARG. Both complexes maintained stable RMSD and Rg values throughout the simulation. Their significantly negative binding free energies further confirmed strong and stable binding. MM-PBSA decomposition and electrostatic potential analysis revealed distinct binding mechanisms between the two systems. The Morin-ESR1 interaction was primarily driven by van der Waals forces, supported by the compound’s stable hydrophobic contacts within the binding pocket. A persistent hydrogen-bond network with GLU353 and ARG394, reflected in consistently short atomic distances (Fig. 8I), further stabilized the complex.

In contrast, Asimilobine-PPARG binding depended more on electrostatic interactions. Although the hydrogen bond with LEU340 was dynamic, strong hydrophobic contacts—evident from shortened atomic distances after equilibration—ensured robust binding. This hydrophobic encapsulation, combined with local electrostatic complementarity, compensated for the fewer hydrogen bonds and maintained high affinity.

The convergence of MD trajectories and the presence of a single deep energy well in FEL confirmed that the docked binding poses represent reliable, low-energy conformations. Together, these simulations not only validate the predicted binding modes but also provide a dynamic and thermodynamic basis for the high-affinity interactions between FH03FS bioactives and their core targets.

## Conclusions

This integrated in silico analysis establishes the potential of FH03FS, a sterilized fermented beverage, for combating obesity and T2D. The beverage is rich in bioactive aporphines and flavonoids with favorable ADMET properties, and its predicted efficacy stems from a multi-target mechanism that concurrently addresses both metabolic and inflammatory processes. The strong binding affinities observed in molecular docking, coupled with the robust stability of representative complexes under MD simulations, provide a computational basis for this multi-target mechanism.

We explicitly acknowledge that the mechanistic insights presented here are derived from computational predictions. While the in silico framework offers valuable insights, it also presents inherent limitations. The 100-ns MD simulations, while confirming short-term structural stability, may not capture slower conformational dynamics. Consequently, the proposed network pharmacology mechanisms and high-affinity interactions remain hypothetical. Future work must therefore employ biophysical assays and functional studies in vitro and in vivo to confirm the bioactivity and therapeutic potential of FH03FS.

Notwithstanding these limitations, this study bridges a critical translational gap by directly characterizing the final, sterilized product, FH03FS. Our work establishes a compelling phytochemical and mechanistic rationale for its development as a multi-target functional beverage and provides a set of rigorously testable hypotheses to guide future in vitro and in vivo validation in the management of metabolic syndromes.

## Supplementary Information

Below is the link to the electronic supplementary material.

Supplementary Material 1

## Data Availability

The molecular dynamics simulation datasets generated during the current study are available in the Zenodo repository, https://doi.org/10.5281/zenodo.17336868.

## Declarations

### Supplementary Information

The supplementary materials are available as a single file, which includes Supplementary Tables S1-S4 and Supplementary Figure S1. These provide supporting evidence for the compound relative quantification in FH03FS and the network pharmacology validation of 20 therapeutic targets.
