# Supplementary material for: Bioactive aporphines and flavonoids from a fermented beverage target metabolic inflammatory pathways in obesity and type 2 diabetes
Source: Sci Rep. 2025 Dec 8;16:1206. doi: 10.1038/s41598-025-30778-9 (PMC12789121; doi:10.1038/s41598-025-30778-9)
Supplement: Supplementary file 1 — Supplementary Material 1 [file 41598_2025_30778_MOESM1_ESM.pdf]

Supplementary Information for

“Bioactive aporphines and flavonoids from a fermented beverage target metabolic inflammatory pathways in obesity and type 2 diabetes”

Xiurong Wu[1], Yang Qiu[1], Zixu Huang[1], Rui Dai[1], Jinghan Wang[1], Xiantao Yan[2*], Xiangzhen Nie[1,3], Ronghan Liu[1,4]

[1] School of Food and Health, Guilin Tourism University, Guilin, China
[2] School of Life Sciences and Food Engineering, Hanshan Normal University, Chaozhou, China
[3] Key Laboratory of Industrialized Processing and Safety of Guangxi Cuisine (Guilin Tourism University), Education Department of Guangxi Zhuang Autonomous Region, Guilin, China
[4] Guangxi Engineering Research Center for Large-Scale Preparation & Nutrients and Hygiene of Guangxi Cuisine, Guilin, China

*Corresponding Author: yanyang214@126.com

Supplemental Figure S1. Total ion flow diagrams of FH03FS samples.

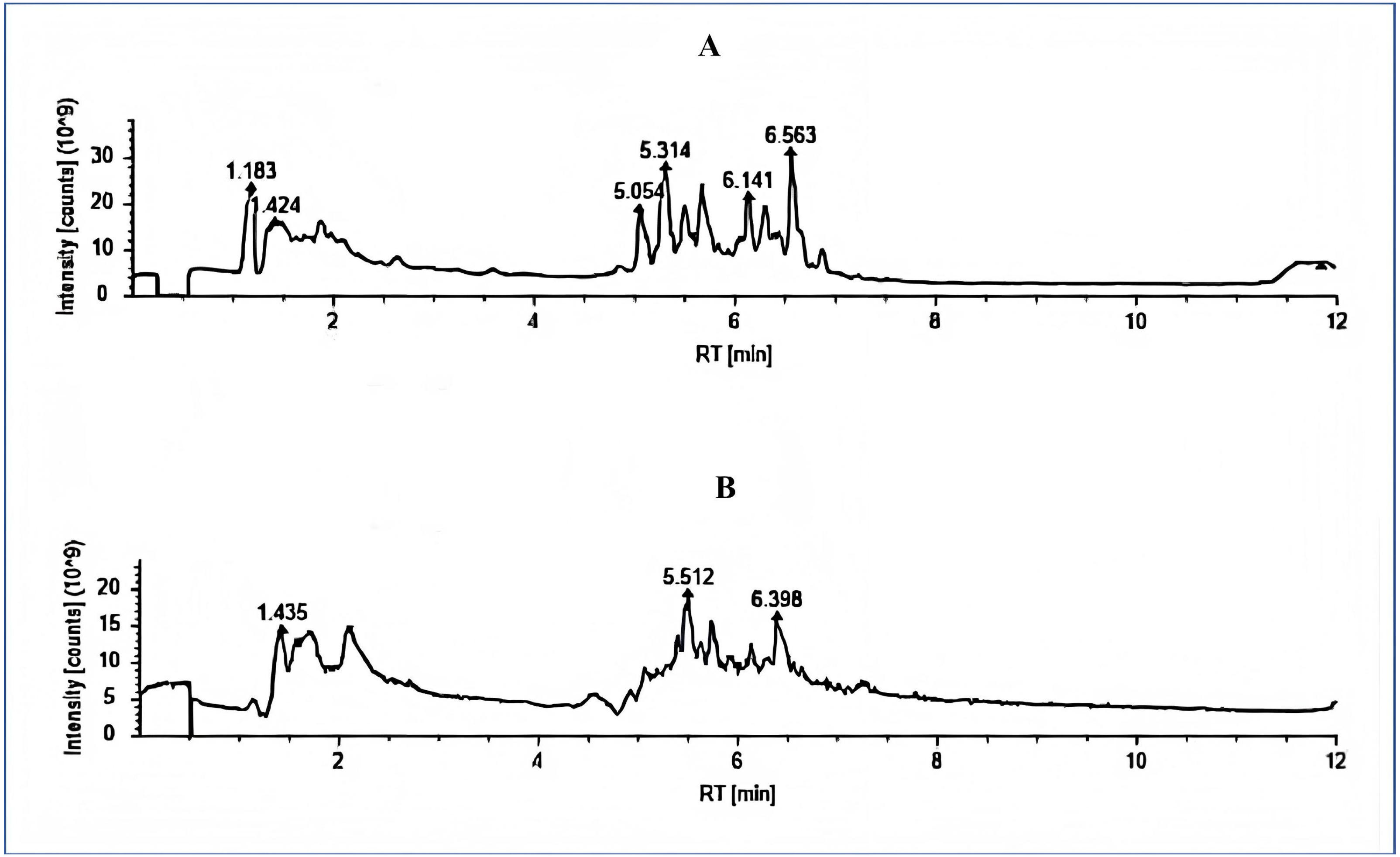

Supplemental Table S1. List of 131 compounds with relative abundance ＞0.1%.

Compound ID	Name	Formula	Molecular Weight	m/z	RT (min)	ClassI	ClassII	SMILES	InChIKey	FH03FS1	FH03FS2	FH03FS3	Relative abundance (%)
Mol 1	Acetylagmatine	$C_7H_{16}N_4O$	172.13241	173.13987	2.287	Organic acids and derivatives	Carboxylic acids and derivatives	CC(=O)NCCCCN=C(N)N	JMACEDIUUMWDIC-UHFFFAOYSA-N	161101000000	155203000000	163323000000	6.499367406
Mol 2	Hexamethylquercetagetin	$C_{21}H_{22}O_8$	402.13147	403.13889	6.641	Phenylpropanoids and polyketides	Flavonoids	O=C1C(OC)=C(OC=2C=C(OC)C(OC)=C(OC)C12)C=3C=CC(OC)=C(OC)C3	CHXSDKWBSFDZEU-UHFFFAOYSA-N	160226000000	149343000000	155951000000	6.308205157
Mol 3	1-Methyl-corypalline	$C_{12}H_{17}NO_2$	207.12593	208.13341	5.147	Organoheterocyclic compounds	Tetrahydroisoquinolines	CC1C2=CC(=C(C=C2CCN1C)OC)O	QFSVLNAGJRAZFV-UHFFFAOYSA-N	138542000000	146097000000	140745000000	5.76432708
Mol 4	Proline betaine	$C_7H_{13}NO_2$	143.09463	144.10199	1.544	Organic acids and derivatives	Carboxylic acids and derivatives	O=C([O-])C1CCC[N+]1(C)C	CMUNUTVVOOHQPW-LURJTMIESA-N	118550000000	122967000000	114299000000	4.821619535
Mol 5	Nuciferine	$C_{19}H_{21}NO_2$	295.15723	296.16474	5.76	Alkaloids and derivatives	Aporphines	CN1CCC2=CC(=C(C3=C2C1CC4=CC=CC=C43)OC)OC	ORJVQPIHKOARKV-OAHLLOKOSA-N	74516884327	74717291319	76245092815	3.055442267
Mol 6	Phenylephrine	$C_9H_{13}NO_2$	167.09463	150.09146	1.847	Benzenoids	Phenols	CNC[C@H](O)C1=CC(O)=CC=C1	SONNWYBIRXJNDC-VIFPVBQESA-N	68656354804	70184079608	67136263895	2.7911653
Mol 7	Hexadecasphinganine	$C_{16}H_{35}NO_2$	273.26678	274.27413	6.856	Organic nitrogen compounds	Organonitrogen compounds	CCCCCCCCCCCCCC(C(CO)N)O	ZKLREJQHRKUJHD-JKSUJKDBSA-N	48839154336	52750983680	53625750156	2.103311708
Mol 8	O-Nornuciferine	$C_{18}H_{19}NO_2$	281.14158	282.14911	5.563	Alkaloids and derivatives	Aporphines	COC1=C(O)C=C2CCN(C)C3CC4=CC=CC=C4C1=C23	AKXOIHNFHOEPHN-UHFFFAOYSA-N	52822206723	46481254621	47216771660	1.985477937
Mol 9	9S,12S,13S-Trihydroxy-10E-octadecenoic acid	$C_{18}H_{34}O_5$	330.24063	329.23342	6.513	Lipids and lipid-like molecules	Fatty Acyls	CCCCCC(C(C=CC(CCCCCCCC(=O)O)O)O)O	MDIUMSLCYIJBQC-MVFSOIOZSA-N	60258673308	27269316407	58256256658	1.975504672
Mol 10	Isosinensetin	$C_{20}H_{20}O_7$	372.1209	373.12833	6.343	Phenylpropanoids and polyketides	Flavonoids	COC1=CC=C(C=C1OC)C1=CC(=O)C2=C(O1)C(OC)=C(OC)C=C2OC	UYCWETIUOAGWIL-UHFFFAOYSA-N	42929028517	41203972280	50921715534	1.830110109

Mol 11	Morin	$C_{15}H_{10}O_7$	302.04265	301.03548	5.954	Phenylpropanoids and polyketides	Flavonoids	OC1=CC(O)=C(C=C1)C1=C(O)C(=O)C2=C(O)C=C(O)C=C2O1	YXOLAZRVSSWPPT-UHFFFAOYSA-N	32699401545	47244014724	48317933910	1.738054027
Mol 12	Indolelactic acid	$C_{11}H_{11}NO_3$	205.07389	188.07081	5.319	Organoheterocyclic compounds	Indoles and derivatives	O[C@@H](CC1=CNC2=C1C=CC=C2)C(O)=O	XGILAAMKEQUXLS-JTQLQIEISA-N	29799398865	31107999516	29662316889	1.227299246
Mol 13	Dehydrojuncusol	$C_{18}H_{16}O_2$	264.11503	265.12243	5.765	Benzenoids	Phenanthrenes and derivatives	CC1=C(C=CC2=C1C=CC3=CC(=C(C(=C32)C=C)C)O)O	IZVFYHBVHNNKGE-UHFFFAOYSA-N	27931591842	28132653972	28313662981	1.143394826
Mol 14	Trigonelline	$C_7H_7NO_2$	137.04768	138.05511	1.46	Alkaloids and derivatives	-	C[N+]1=CC=CC(=C1)C([O-])=O	WWNNZCOKKKDOPX-UHFFFAOYSA-N	27409412117	33331145145	20282973040	1.097940049
Mol 15	Hypaphorine	$C_{14}H_{18}N_2O_2$	246.13683	247.14438	5.306	Organic acids and derivatives	Carboxylic acids and derivatives	C[N+](C)(C)C(CC1=CNC2=CC=CC=C21)C(=O)[O-]	AOHCBEAZXHZMOR-ZDUSSCGKSA-N	25393215588	26356552137	26056364635	1.054341479
Mol 16	Adenine monohydrochloride hemihydrate	$C_5H_5N_5$	135.0545	136.06192	1.718	Organic acids and derivatives	Carboxylic acids and derivatives	C1=NC2=NC=NC(=C2N1)N.O.Cl	DHMQDGOQFOQNFH-UHFFFAOYSA-N	25253311799	26498440095	24653623271	1.035359988
Mol 17	(Z)-5,8,11-trihydroxyoctadec-9-enoic acid	$C_{18}H_{34}O_5$	330.24063	353.23003	7.129	Lipids and lipid-like molecules	Fatty Acyls	O=C(O)CCCC(O)CCC(O)C=CC(O)CCCCCCC	FITAKYVPWCVTII-QBFSEMIESA-N	22133831965	26040164267	26566090578	1.012793867
Mol 18	Miquelianin	$C_{21}H_{18}O_{13}$	478.07475	477.06765	5.611	Phenylpropanoids and polyketides	Flavonoids	OC1C(O)C(OC2=C(OC3=CC(O)=CC(O)=C3C2=O)C2=CC=C(O)C(O)=C2)OC(C1O)C(O)=O	DUBCCGAQYVUYEU-UHFFFAOYSA-N	23850944692	26120356885	22279623075	0.979063532
Mol 19	Armepavine	$C_{19}H_{23}NO_3$	313.16779	314.17522	5.375	Organoheterocyclic compounds	Isoquinolines and derivatives	COC1=CC2=C(C=C1OC)C(CC1=CC=C(O)C=C1)N(C)CC2	ZBKFZIUKXTWQTP-UHFFFAOYSA-N	23371868351	21850171267	23137748929	0.926335218
Mol 20	L-Phenylalanine	$C_9H_{11}NO_2$	165.07898	166.08642	5.04	Organic acids and derivatives	Carboxylic acids and derivatives	N[C@@H](CC1=CC=CC=C1)C(O)=O	COLNVLDHVKWLRT-QMMMGPOBSA-N	21018274908	22655760268	21321451868	0.880745976

Mol 21	Quinic acid	$C_7H_{12}O_6$	192.06339	191.0559	1.442	Organic oxygen compounds	Organooxygen compounds	O[C@@H]1C[C@@](O)(C[C@@H](O)[C@H]1O)C(O)=O	AAWZDTNXLSGCEK-WYWMIBKRSA-N	19559148883	23331517537	16481929651	0.804550861
Mol 22	L-Pipecolic acid	$C_6H_{11}NO_2$	129.07898	130.08642	1.75	Organic acids and derivatives	Carboxylic acids and derivatives	OC(=O)[C@@H]1CCCCN1	HXEACLLIILLPRG-YFKPBYRVSA-N	19834362759	20547042281	18951019838	0.804006506
Mol 23	5-oxoproline	$C_5H_7NO_3$	129.04259	130.05006	2.199	Organic acids and derivatives	Carboxylic acids and derivatives	C1CC(=O)NC1C(=O)O	ODHCTXKNWHHXJC-UHFFFAOYSA-N	18703041857	20133493350	18947875807	0.783029557
Mol 24	Corchorifatty acid F	$C_{18}H_{32}O_5$	328.22498	327.21808	6.504	Lipids and lipid-like molecules	Fatty Acyls	CCC=CCC(C(C=CC(CCCCCCCC(=O)O)O)O)O	MKYUCBXUUSZMQB-MKZMYESJSA-N	19499783877	18797637930	18330418264	0.767357005
Mol 25	(S)-N-Methylcoclaurine	$C_{18}H_{21}NO_3$	299.15214	300.15982	5.367	Organoheterocyclic compounds	Isoquinolines and derivatives	CN1CCC2=CC(=C(C=C2C1CC3=CC=C(C=C3)O)O)OC	BOKVLBSSPUTWLV-INIZCTEOSA-N	18504987469	18346289404	19430332569	0.762665275
Mol 26	Roemerine	$C_{18}H_{17}NO_2$	279.12593	280.13346	5.78	Alkaloids and derivatives	Aporphines	CN1CCC2=CC3=C(OCO3)C3=C2C1CC1=CC=CC=C31	JCTYWRARKVGOBK-UHFFFAOYSA-N	15188003441	13268636611	13810139551	0.572752013
Mol 27	(9Z,12E)-15,16-dihydroxyoctadeca-9,12-dienoic acid	$C_{18}H_{32}O_4$	312.23006	335.21955	7.313	Lipids and lipid-like molecules	Fatty Acyls	O=C(O)CCCCCCCC=CCC=CCC(O)C(O)CC	LKLLJYJTYPVCID-JVVXYUKTSA-N	13024823224	13663539487	15021303332	0.565202635
Mol 28	5,7,3',4'-Tetramethoxyflavone	$C_{19}H_{18}O_6$	342.11034	343.11763	6.602	Phenylpropanoids and polyketides	Flavonoids	COC1=CC2=C(C(=O)C=C(O2)C2=CC=C(OC)C(OC)=C2)C(OC)=C1	CLXVBVLQKLQNRQ-UHFFFAOYSA-N	13790613897	12454460906	14496431261	0.552083216
Mol 29	9-Oxo-10,12-octadecadienoic acid	$C_{18}H_{30}O_3$	294.21949	295.22695	7.299	Lipids and lipid-like molecules	Fatty Acyls	CCCCCC=CC=CC(=O)CCCCCCCC(=O)O	LUZSWWYKKLTDHU-SIGMCMEVSA-N	13513465940	12838896080	14011466771	0.546965357
Mol 30	KOJIC ACID	$C_6H_6O_4$	142.02661	143.03414	2.812	Organoheterocyclic compounds	Pyrans	C1=C(OC=C(C1=O)O)CO	BEJNERDRQOWKJM-UHFFFAOYSA-N	13292656861	13237732529	13745938623	0.545779644

Mol 31	Glucose 3-hydroxybutyrate	$C_{10}H_{18}O_8$	266.10017	265.09302	4.321	Organic oxygen compounds	Organooxygen compounds	CCC(O)C(=O)OC(C(O)C=O)C(O)C(O)CO	HIBYJNNRFVMMRN-UHFFFAOYSA-N	12960512774	12764815467	13804662434	0.535666118
Mol 32	Dehydroeffusol	$C_{17}H_{14}O_2$	250.09938	251.10682	5.58	Benzenoids	Phenanthrenes and derivatives	CC1=C(C=CC2=C1C=CC3=CC(=CC(=C32)C=C)O)O	GSSPKCPIRDPBQE-UHFFFAOYSA-N	12789552853	12770573187	12858434657	0.520605264
Mol 33	3',4',5',5,7-Pentamethoxyflavone	$C_{20}H_{20}O_7$	372.1209	395.11002	6.956	Phenylpropanoids and polyketides	Flavonoids	COC1=CC(OC)=C2C(=O)C=C(OC2=C1)C1=CC(OC)=C(OC)C(OC)=C1	GIKVSFNAEBQLGB-UHFFFAOYSA-N	13285210612	12585619210	12095094097	0.514471638
Mol 34	Phenyllactic acid	$C_9H_{10}O_3$	166.063	165.05547	5.81	Phenylpropanoids and polyketides	Phenylpropanoic acids	OC(CC1=CC=CC=C1)C(O)=O	VOXXWSYKYCBWHO-UHFFFAOYSA-N	12099227564	12950816129	11677558744	0.497691294
Mol 35	Cepharatine B	$C_{18}H_{19}NO_4$	313.13141	314.13898	5.183	Benzenoids	Phenanthrenes and derivatives	CN1CCC23CC1(C(=O)C=C2C=CC4=CC(=C(C=C34)OC)O)O	LBRUFSNOGWAARZ-MSOLQXFVSA-N	11827476965	11241145802	10663844267	0.457104577
Mol 36	Neoamphimedine	$C_{19}H_{11}N_3O_2$	313.08513	314.09215	2.706	Organoheterocyclic compounds	Quinolines and derivatives	CN1C=CC2=C(C1=O)C(=O)C3=NC=CC4=C3C2=NC5=CC=CC=C45	HHTUKGISODJJKH-UHFFFAOYSA-N	11265473623	10713150827	11707305677	0.456473961
Mol 37	2-C-methyl-D-erythritol-4-phosphate	$C_5H_{13}O_7P$	216.03989	215.03276	1.4	Organic acids and derivatives	Organic phosphoric acids and derivatives	[H][C@@](O)(COP(O)(O)=O)[C@@](C)(O)CO	XMWHRVNVKDKBRG-UHNVWZDZSA-N	10266757595	11555443106	10149472249	0.433244269
Mol 38	Homoisocitrate	$C_7H_{10}O_7$	206.04265	205.03515	1.477	Organic acids and derivatives	Carboxylic acids and derivatives	C(CC(=O)O)C(C(C(=O)O)O)C(=O)O	OEJZZCGRGVFWHK-WVZVXSGGSA-N	10643278009	11165129522	9350240096	0.422227061
Mol 39	L-Malate	$C_4H_6O_5$	134.02152	133.01412	1.572	Organic acids and derivatives	Hydroxy acids and derivatives	O[C@@H](CC(O)=O)C(O)=O	BJEPYKJPYRNKOW-REOHCLBHSA-N	10272250243	10892345164	9782223119	0.419356591
Mol 40	9,10-Epoxy-13-hydroxy-11-octadecenoate	$C_{18}H_{32}O_4$	312.23006	313.23738	7.132	Organic acids and derivatives	Hydroxy acids and derivatives	CCCCCC(C=CC1C(O1)CCCCCCCC(=O)O)O	BWLQUNFALXKBSJ-BUHFOSPRSA-N	9056835516	11364877414	9935051249	0.411360836

Mol 41	1-Hydroxy-6-methoxypyrene	$C_{17}H_{12}O_2$	248.08373	249.09128	5.8	Benzenoids	Pyrenes	COC1=C2C=CC3=C4C2=C(C=CC4=C(C=C3)O)C=C1	BVDKRJLKWHAGMW-UHFFFAOYSA-N	9842229111	9687815188	10058993873	0.400957474
Mol 42	PISCIDIC ACID	$C_{11}H_{12}O_7$	256.05831	255.05113	5.075	Phenylpropanoids and polyketides	Phenylpropanoic acids	C1=CC(=CC=C1CC(C(C(=O)O)O)(C(=O)O)O)O	TUODPMGCCJSJRH-UHFFFAOYSA-N	9449807254	9529671351	9489689853	0.385782255
Mol 43	Adenosine	$C_{10}H_{13}N_5O_4$	267.09676	268.10438	2.999	Nucleosides, nucleotides, and analogues	Purine nucleosides	NC1=C2N=CN([C@@H]3O[C@H](CO)[C@@H](O)[C@H]3O)C2=NC=N1	OIRDTQYFTABQOQ-KQYNXXCUSA-N	9355479692	8162082934	8832507168	0.357066605
Mol 44	(S)-Coclaurine	$C_{17}H_{19}NO_3$	285.13649	286.14408	5.503	Organoheterocyclic compounds	Isoquinolines and derivatives	COC1=C(C=C2C(NCCC2=C1)CC3=CC=C(C=C3)O)O	LVVKXRQZSRUVPY-HNNXBMFYSA-N	8621691951	8667703727	7314919788	0.3334101
Mol 45	9-Hydroxy-10E,12Z-octadecadienoic acid	$C_{18}H_{32}O_3$	296.23514	297.24248	6.536	Lipids and lipid-like molecules	Fatty Acyls	CCCCCC=CC=CC(CCCCCCCC(=O)O)O	NPDSHTNEKLQQIJ-ZJHFMPGASA-N	8059737172	8755283804	7599790854	0.330842159
Mol 46	GLYCERATE	$C_3H_6O_4$	106.02661	105.01918	1.425	Organic oxygen compounds	Organooxygen compounds	O=C(O)C(O)CO	RBNPOMFGQQGHHO-UWTATZPHSA-N	8945273832	8799962797	6018210582	0.322015596
Mol 47	3'-Demethylnobiletin	$C_{20}H_{20}O_8$	388.11582	389.12305	6.358	Phenylpropanoids and polyketides	Flavonoids	COC1=C(O)C=C(C=C1)C1=CC(=O)C2=C(O1)C(OC)=C(OC)C(OC)=C2OC	XFYYZBJXMSDKCV-UHFFFAOYSA-N	7459586800	7197573747	8870992148	0.318827148
Mol 48	Bellendine	$C_{12}H_{15}NO_2$	205.11028	206.1179	5.082	Organoheterocyclic compounds	Cycloheptapyrans	CC1=COC2=C(C1=O)C3CCC(C2)N3C	ODQUOOUDMCGJCX-UHFFFAOYSA-N	7861112814	7711919228	7952756509	0.318795112
Mol 49	Limonenecarboxylic acid	$C_{11}H_{16}O_2$	180.11503	181.12233	6.295	Lipids and lipid-like molecules	Prenol lipids	CC1=CCC(CC1)C(=CC(=O)O)C	KENWRRUSJKMXOL-PCYYEKQGSA-N	7288749967	8391175815	7523198949	0.314422734
Mol 50	Guanine	$C_5H_5N_5O$	151.04941	152.05682	3.295	Organoheterocyclic compounds	Imidazopyrimidines	N=C1N=C(O)C=2N=CNC2N1	UYTPUPDQBNUYGX-UHFFFAOYSA-N	7632253543	7209427304	8126393308	0.311237592

Mol 51	(R)-Leucic acid	$C_6H_{12}O_3$	132.07864	131.07118	5.797	Lipids and lipid-like molecules	Fatty Acyls	CC(C)C[C@@H](O)C(O)=O	LVRFTAZAXQPQHI-RXMQYKEDSA-N	7158452951	7776214723	7062198834	0.298076875
Mol 52	N-(1-Deoxy-1-fructosyl)proline	$C_{11}H_{19}NO_7$	277.11615	278.12374	1.573	Organic acids and derivatives	Carboxylic acids and derivatives	OC[C@H]1OC(O)(CN2CCCC2C(O)=O)[C@@H](O)[C@@H]1O	GSFRYAKUDPPHHG-MKSMHYHASA-N	7254963349	7434644216	7148804086	0.295929672
Mol 53	Citraconic acid	$C_5H_6O_4$	130.02661	111.00868	2.094	Lipids and lipid-like molecules	Fatty Acyls	C\C(=C\C(O)=O)C(O)=O	HNEGQIOMVPPMNR-IHWYPQMZSA-N	5599892219	9887724070	5406971699	0.283140032
Mol 54	9,10-DiHOME	$C_{18}H_{34}O_4$	314.24571	313.23857	7.422	Lipids and lipid-like molecules	Fatty Acyls	CCCCCC=CCC(C(CCCCCCCC(=O)O)O)O	XEBKSQSGNGRGDW-YFHOEESVSA-N	6059912178	5380455002	9408792690	0.282524441
Mol 55	9R-Hydroxy-10E,12Z,15Z-Octadecatrienoic acid	$C_{18}H_{30}O_3$	294.21949	295.22687	6.489	Lipids and lipid-like molecules	Fatty Acyls	CCC=CCC=CC=CC(CCCCCCCC(=O)O)O	RIGGEAZDTKMXSI-RWUWUJKWSA-N	6444597316	7167001360	6618810049	0.274139819
Mol 56	L-Norleucine	$C_6H_{13}NO_2$	131.09463	114.09154	2.3	Organic acids and derivatives	Carboxylic acids and derivatives	CCCC[C@H](N)C(O)=O	LRQKBLKVPFOOQJ-YFKPBYRVSA-N	6591872802	6323394344	6664205683	0.265319066
Mol 57	9,10-12,13-Diepoxyoctadecanoate	$C_{18}H_{32}O_4$	312.23006	311.2231	7.311	Lipids and lipid-like molecules	Fatty Acyls	CCCCCC1C(O1)CC2C(O2)CCCCCCCC(=O)O	LAIUZQCFIABNDP-UHFFFAOYSA-N	6234531630	6332828253	6664146453	0.260603814
Mol 58	Eudesmin	$C_{22}H_{26}O_6$	386.17294	409.16227	6.53	Lignans, neolignans and related compounds	Furanoid lignans	COC1=C(C=C(C=C1)C2C3COC(C3CO2)C4=CC(=C(C=C4)OC)OC)OC	PEUUVVGQIVMSAW-RZTYQLBFSA-N	6599859625	6885856825	5337830873	0.255075611
Mol 59	Sucralose	$C_{12}H_{19}Cl_3O_8$	396.01455	395.00741	5.541	Organic oxygen compounds	Organooxygen compounds	ClCC1OC(OC2OC(CO)C(Cl)C(O)C2O)(CCl)C(O)C1O	BAQAVOSOZGMPRM-QBMZZYIRSA-N	5948355696	6384563846	5479242847	0.241370456
Mol 60	Tianshic acid methylester	$C_{19}H_{36}O_5$	344.25628	343.24891	7.122	Lipids and lipid-like molecules	Fatty Acyls	CCCCCCCC(C(C=CC(CCCCCCC(=O)OC)O)O)O	VAGGDCACZPJMFS-CCEZHUSRSA-N	6438819896	5866360962	5463288940	0.240778383

Mol 61	D-1-[(3-Carboxypropyl)amino]-1-deoxyfructose	$C_{10}H_{19}NO_7$	265.11615	266.12372	1.476	Organic acids and derivatives	Carboxylic acids and derivatives	OC[C@H]1O[C@](O)(CNCCCC(O)=O)[C@@H](O)[C@@H]1O	HUEOABWGBTXQNF-SFKDOBOXSA-N	5520457029	6029835995	5220870935	0.227264012
Mol 62	N-Fructosyl isoleucine	$C_{12}H_{23}NO_7$	293.14745	276.1445	3.854	Organic acids and derivatives	Carboxylic acids and derivatives	O=C(O)C(NCC1(O)OC(CO)C(O)C1O)C(C)CC	VYGRYVGDPYFVCA-UHFFFAOYSA-N	5696340551	5736361707	5067984648	0.22359881
Mol 63	BETAMIPRON	$C_{10}H_{11}NO_3$	193.07389	194.08147	2.858	Benzenoids	Benzene and substituted derivatives	C1=CC=C(C=C1)C(=O)NCCC(=O)O	CWXYHOHYCJXYFQ-UHFFFAOYSA-N	5493976923	5347248020	5487578297	0.221269636
Mol 64	(-)-Aristeromycin	$C_{11}H_{15}N_5O_3$	265.11749	266.12575	5.765	Nucleosides, nucleotides, and analogues	Nucleoside and nucleotide analogues	C1C(C(C(C1N2C=NC3=C(N=CN=C32)N)O)O)CO	UGRNVLGKAGREKS-GCXDCGAKSA-N	5313959618	5383025541	5276801893	0.216458854
Mol 65	Betonicine	$C_7H_{13}NO_3$	159.08954	160.09696	1.448	Organic acids and derivatives	Carboxylic acids and derivatives	O=C([O-])C1CC(O)C[N+]1(C)C	MUNWAHDYFVYIKH-RITPCOANSA-N	5074878229	5936290064	4952858543	0.216326595
Mol 66	2-amino-1,3,4,5-icosanetetrol	$C_{20}H_{43}NO_4$	361.31921	362.3266	6.857	Organic nitrogen compounds	Organonitrogen compounds	CCCCCCCCCCCCCCCC(C(C(C(CO)N)O)O)O	ILMFJEORWZZXPC-UHFFFAOYSA-N	5078187608	5131860867	5727882997	0.21597298
Mol 67	Higenamine	$C_{16}H_{17}NO_3$	271.12084	272.12839	5.151	Organoheterocyclic compounds	Isoquinolines and derivatives	OC1=CC=C(CC2NCCC3=C2C=C(O)C(O)=C3)C=C1	WZRCQWQRFZITDX-UHFFFAOYSA-N	5240052054	5400102915	5281496212	0.215752367
Mol 68	Indole	C_8H_7N	117.05785	118.06526	5.31	Organoheterocyclic compounds	Indoles and derivatives	N1C=CC2=C1C=CC=C2	SIKJAQJRHWYJAI-UHFFFAOYSA-N	5068218640	5542991320	5126199101	0.213255725
Mol 69	Vasicolinone	$C_{19}H_{19}N_3O$	305.15281	306.15976	5.203	Organoheterocyclic compounds	Diazanaphthalenes	CN(C)C1=CC=CC=C1C2CCN3C2=NC4=CC=CC=C4C3=O	ADHMTMIOMYKZHH-UHFFFAOYSA-N	5327936648	5084945481	5062480689	0.209704768
Mol 70	Ephedroxane	$C_{11}H_{13}NO_2$	191.09463	192.10218	4.875	Organoheterocyclic compounds	Azolidines	CC1C(OC(=O)N1C)C2=CC=CC=C2	MNYARIILPGRTQL-WPRPVWTQSA-N	5083928735	5112876200	5256636550	0.209407715

Mol 71	Indole-3-carboxaldehyde	C_9H_7NO	145.05276	146.06016	5.32	Organoheterocyclic compounds	Indoles and derivatives	O=CC1=CNC2=C1C=CC=C2	OLNJUISKUQQNIM-UHFFFAOYSA-N	5096936236	4855141471	5083051571	0.203739216
Mol 72	O-acryloyl-L-carnitine	$C_{10}H_{17}NO_4$	215.11576	216.12334	1.604	Lipids and lipid-like molecules	Fatty Acyls	C[N+](C)(C)CC(CC(=O)[O-])OC(=O)C=C	YUCNWOKTRWJLGY-MRVPVSSYSA-N	5827873703	4559482917	4393329082	0.200291282
Mol 73	Linamarin	$C_{10}H_{17}NO_6$	247.10559	248.11317	1.476	Organic oxygen compounds	Organooxygen compounds	CC(C)(C#N)OC1C(C(C(C(O1)CO)O)O)O	QLTCHMYAEJEXBT-ZEBDFXRSSA-N	4787184862	5278130942	4661731458	0.199564434
Mol 74	Sorbitan laurate	$C_{18}H_{34}O_6$	346.23554	345.22808	6.511	Lipids and lipid-like molecules	Fatty Acyls	CCCCCCCCCCCC(=O)OCC(O)C1OCC(O)C1O	LWZFANDGMFTDAV-UHFFFAOYSA-N	4871209123	5611203050	3811541462	0.193695635
Mol 75	2-Hydroxy-3-methylbutyric acid	$C_5H_{10}O_3$	118.063	117.05559	5.428	Lipids and lipid-like molecules	Fatty Acyls	CC(C)C(O)C(O)=O	NGEWQZIDQIYUNV-UHFFFAOYSA-N	4711360299	4791669088	4675699610	0.19213424
Mol 76	Stemofuran K	$C_{18}H_{18}O_3$	282.12559	283.13324	5.404	Phenylpropanoids and polyketides	2-arylbenzofuran flavonoids	CC1=C(C(=C(C(=C1O)C)OC)C)C2=CC3=CC=CC=C3O2	GNIVXBUUCHPMOA-UHFFFAOYSA-N	4763104733	4600108043	4796258655	0.191873283
Mol 77	Threonic acid	$C_4H_8O_5$	136.03717	135.02974	1.413	Organic oxygen compounds	Organooxygen compounds	OC[C@H](O)[C@@H](O)C(O)=O	JPIJQSOTBSSVTP-STHAYSLISA-N	4558681771	5465574562	3964117270	0.189554757
Mol 78	2-Hydroxycinnamic acid	$C_9H_8O_3$	164.04735	147.04415	5.916	Phenylpropanoids and polyketides	Cinnamic acids and derivatives	OC(=O)\C=C\C1=C(O)C=CC=C1	PMOWTIHVNWZYFI-AATRIKPKSA-N	4691307302	4953160999	4212589976	0.187775319
Mol 79	Arginine propyl ester	$C_9H_{20}N_4O_2$	216.15863	217.16624	3.155	Organic acids and derivatives	Carboxylic acids and derivatives	O=C(OCCC)C(N)CCCNC(=N)N	HJMAILFRPWZGMI-UHFFFAOYSA-N	4422193770	4292183202	4362245351	0.177199726
Mol 80	5-O-Demethylnobiletin	$C_{20}H_{20}O_8$	388.11582	389.12313	6.884	Phenylpropanoids and polyketides	Flavonoids	COC1=C(OC)C=C(C=C1)C1=CC(=O)C2=C(O)C(OC)=C(OC)C(OC)=C2O1	DOFJNFPSMUCECH-UHFFFAOYSA-N	4667838215	4109875800	4255262083	0.176608281

Mol 81	Potassium 2-hydroxy-2-methylsuccinate	$C_5H_8O_5$	148.03717	147.0298	2.755	-	-	CC(CC(=O)[O-])(C(=O)[O-])O.[K+].[K+]	VCJUJDUIBZISHE-UHFFFAOYSA-L	4331069462	4111017999	4295931858	0.172611358
Mol 82	p-Octopamine	$C_8H_{11}NO_2$	153.07898	136.07587	2.49	Benzenoids	Phenols	NC[C@H](O)C1=CC=C(O)C=C1	QHGUCRYDKWKLMG-QMMMGPOBSA-N	4057204077	4013055463	4233275002	0.166723707
Mol 83	cytosine	$C_4H_5N_3O$	111.04326	112.05069	1.644	Organoheterocyclic compounds	Diazines	N=C1C=CN=C(O)N1	OPTASPLRGRRNAP-UHFFFAOYSA-N	4415653648	4689087674	2938409681	0.163195281
Mol 84	2,2,6,7-Tetramethylbicyclo[4.3.0]nona-1(9),4-dien	$C_{13}H_{18}O$	190.13576	191.14314	6.145	Organic oxygen compounds	Organooxygen compounds	CC1C(=O)C=C2C1(C=CCC2(C)C)C	KTDAEZJBJUWAPC-UHFFFAOYSA-N	4539326562	4020736004	3463760828	0.162933375
Mol 85	Oleamide	$C_{18}H_{35}NO$	281.27186	304.2612	9.928	Lipids and lipid-like molecules	Fatty Acyls	CCCCCCCC\C=C/CCCCCCCC(N)=O	FATBGEAMYMYZAF-KTKRTIGZSA-N	8064193657	1619928016	1983318697	0.158104072
Mol 86	3-Keto-beta-ionone	$C_{13}H_{18}O_2$	206.13068	207.1381	6.213	Lipids and lipid-like molecules	Prenol lipids	CC1=C(C(CCC1=O)(C)C)C=CC(=O)C	OBHGOXFSRVNKBS-UHFFFAOYSA-N	3206531608	4311213224	4131482879	0.157857274
Mol 87	Thebaine	$C_{19}H_{21}NO_3$	311.15214	312.15983	5.337	Alkaloids and derivatives	Morphinans	CN1CCC23C4C(=CC=C2C1CC5=C3C(=C(C=C5)OC)O4)OC	FQXXSQDCDRQNQE-VMDGZTHMSA-N	3889450968	3790131593	3968390864	0.157840278
Mol 88	N-Acetyl-L-glutamic acid	$C_7H_{11}NO_5$	189.06372	188.05656	2.348	Organic acids and derivatives	Carboxylic acids and derivatives	CC(=O)N[C@@H](CCC(O)=O)C(O)=O	RFMMMVDNIPUKGG-YFKPBYRVSA-N	3830228775	3919851854	3815165751	0.156719253
Mol 89	Pirbuterol	$C_{12}H_{20}N_2O_3$	240.14739	241.155	2.652	Organoheterocyclic compounds	Pyridines and derivatives	CC(C)(C)NCC(C1=NC(=C(C=C1)O)CO)O	VQDBNKDJNJQRDG-UHFFFAOYSA-N	2549107648	2922947923	5899589015	0.154095779
Mol 90	D-Gluconic Acid	$C_6H_{12}O_7$	196.05831	195.05074	1.439	Organic oxygen compounds	Organooxygen compounds	OC[C@@H](O)[C@@H](O)[C@H](O)[C@@H](O)C(O)=O	RGHNJXZEOKUKBD-SQOUGZDYSA-N	3591237612	4105797075	3426911266	0.150739245

Mol 91	Loliolide	$C_{11}H_{16}O_3$	196.10995	197.11742	5.82	Organoheterocyclic compounds	Benzofurans	O=C1OC2(C(=C1)C(C)(C)CC(O)C2)C	XEVQXKKKAVVSMW-UHFFFAOYSA-N	3658010377	4063524623	3330785985	0.149768664
Mol 92	Asimilobine	$C_{17}H_{17}NO_2$	267.12593	268.13344	5.609	Alkaloids and derivatives	Aporphines	COC1=C(C=C2CCNC3C2=C1C4=CC=CC=C4C3)O	NBDNEUOVIJYCGZ-CYBMUJFWSA-N	3596092119	3720234087	3682399592	0.149042402
Mol 93	Spinosine	$C_{19}H_{21}NO_4$	327.14706	328.15461	5.304	Alkaloids and derivatives	Protoberberine alkaloids and derivatives	COC1=C(C=C2C3CC4=CC(=C(C=C4CN3CCC2=C1)O)O)OC	VAKIESMDOCVMDV-HNNXBMFYSA-N	3572002406	3649631649	3748422309	0.148653906
Mol 94	9,10,18-Trihydroxyoctadecanoic acid	$C_{18}H_{36}O_5$	332.25628	333.26367	6.542	Lipids and lipid-like molecules	Fatty Acyls	C(CCCCO)CCCC(C(CCCCCCCC(=O)O)O)O	OISFHODBOQNZAG-UHFFFAOYSA-N	3543157615	3874821229	3524421901	0.148279148
Mol 95	7,4'-Di-O-methylapigenin	$C_{17}H_{14}O_5$	298.08413	299.09154	6.417	Phenylpropanoids and polyketides	Flavonoids	COC1=CC=C(C=C1)[C@H]2CC(=O)C3=C(C=C(C=C3O2)OC)O	CKEXCBVNKRHAMX-OAHLLOKOSA-N	3511416946	3447769884	3964233979	0.148021953
Mol 96	Oxoadipic acid	$C_6H_8O_5$	160.03717	143.03409	5.348	Organic acids and derivatives	Keto acids and derivatives	OC(=O)CCCC(=O)C(O)=O	FGSBNBBHOZHUBO-UHFFFAOYSA-N	3668950260	3871197017	3334007568	0.147354356
Mol 97	alpha-D-Sedoheptulopyranose 7-phosphate	$C_7H_{15}O_{10}P$	290.04029	291.048	1.621	Organic oxygen compounds	Organooxygen compounds	C(C1C(C(C(C(O1)(CO)O)O)O)O)OP(=O)(O)O	CBIDVWSRUUODHL-OVHBTUCOSA-N	3760262989	3487346305	3470518540	0.145240053
Mol 98	Mallotophenone	$C_{21}H_{24}O_8$	404.14712	405.15454	6.324	Benzenoids	Benzene and substituted derivatives	CC1=C(C(=C(C(=C1OC)CC2=C(C(=C(C(=C2O)C(=O)C)O)C)OC)O)C(=O)C)O	JXSPGOIAWPYMGS-UHFFFAOYSA-N	3375235308	3559269175	3765476640	0.144994149
Mol 99	Germacrone	$C_{15}H_{22}O$	218.16706	219.17447	6.526	Lipids and lipid-like molecules	Prenol lipids	CC(C)=C1CC=C(C)CCC=C(C)CC1=O	CAULGCQHVOVVRN-UHFFFAOYSA-N	4055003807	3268246992	3317607143	0.144192978
Mol 100	1,3-Dimethyluracil	$C_6H_8N_2O_2$	140.05858	158.0926	2.338	Organoheterocyclic compounds	Diazines	CN1C=CC(=O)N(C)C1=O	JSDBKAHWADVXFU-UHFFFAOYSA-N	4061777866	3843558514	2710029802	0.143847542

Mol 101	3,3',4',5,6,7,8-heptamethoxyflavone	$C_{22}H_{24}O_9$	432.14203	455.13117	6.727	Phenylpropanoids and polyketides	Flavonoids	COC1=C(OC)C=C(C=C1)C1=C(OC)C(=O)C2=C(OC)C(OC)=C(OC)C(OC)=C2O1	SSXJHQZOHUYEGD-UHFFFAOYSA-N	3416290651	3697966669	3046291285	0.137684364
Mol 102	1-Isopropyl citrate	$C_9H_{14}O_7$	234.07396	235.0815	1.846	Organic acids and derivatives	Carboxylic acids and derivatives	CC(C)OC(=O)CC(O)(CC(O)=O)C(O)=O	SKHXHUZZFVMERR-UHFFFAOYSA-N	3729339143	2970964858	3424062661	0.137194067
Mol 103	Pyridoxine	$C_8H_{11}NO_3$	169.07389	170.08138	2.189	Organoheterocyclic compounds	Pyridines and derivatives	CC1=C(O)C(CO)=C(CO)C=N1	LXNHXLLTXMVWPM-UHFFFAOYSA-N	3353767620	3295014761	3286614422	0.134633359
Mol 104	Crepenynic acid	$C_{18}H_{30}O_2$	278.22458	279.23204	7.429	Lipids and lipid-like molecules	Fatty Acyls	CCCCCC#CCC=CCCCCCCCC(=O)O	SAOSKFBYQJLQOS-KTKRTIGZSA-N	3130569205	2756357460	3766881013	0.130817579
Mol 105	Pro Ile	$C_{11}H_{20}N_2O_3$	228.14739	229.15502	2.617	Organic acids and derivatives	Carboxylic acids and derivatives	CCC(C)C(C(=O)O)NC(=O)C1CCCN1	OCYROESYHWUPBP-VGMNWLOBSA-N	2423641986	3636500535	3503742304	0.129599045
Mol 106	Isocitrate	$C_6H_8O_7$	192.02701	173.00893	1.567	Organic acids and derivatives	Carboxylic acids and derivatives	OC(C(CC(O)=O)C(O)=O)C(O)=O	ODBLHEXUDAPZAU-UHFFFAOYSA-N	3151976850	3498600039	2913200163	0.129597585
Mol 107	Gulonolactone	$C_6H_{10}O_6$	178.04774	177.04036	2.388	Organoheterocyclic compounds	Lactones	[H][C@@]1(OC(=O)[C@@H](O)[C@H]1O)[C@@H](O)CO	SXZYCXMUPBBULW-SKNVOMKLSA-N	3154278083	3080734666	3253977691	0.128584161
Mol 108	Tashironin	$C_{22}H_{26}O_6$	386.17294	387.18033	6.532	Benzenoids	Benzene and substituted derivatives	CC1CCC2(C13CC(=O)C4(C2(COC4(C3O)OC(=O)C5=CC=CC=C5)C)C)O	BRRTVYWNYBLCAX-JHMPXYSCSA-N	3359748446	3230072169	2840813888	0.127793386
Mol 109	Glucose propionate	$C_9H_{16}O_8$	252.08452	251.07722	1.624	Organic oxygen compounds	Organooxygen compounds	CCC(=O)OC1(O)OC(CO)C(O)C(O)C1O	PCFIAZZAJNYGGV-UHFFFAOYSA-N	3124147765	3676726411	2604502327	0.127451118
Mol 110	4,4-Difluoropregn-5-ene-3,20-dione	$C_{21}H_{28}F_2O_2$	350.20574	351.21394	7.228	Lipids and lipid-like molecules	Steroids and steroid derivatives	CC(=O)C1CCC2C1(CCC3C2CC=C4C3(CCC(=O)C4(F)F)C)C	OTTUKNSDVUSPGW-WOEIGVFJSA-N	3062720017	3236161776	2939560678	0.125189015

Mol 111	N1,N8-Diacetylspermidine	$C_{11}H_{23}N_3O_2$	229.17903	230.18666	2.299	Organic acids and derivatives	Carboxylic acids and derivatives	CC(=O)NCCCCNCCCNC(C)=O	BKCVMAZDKFQPHB-UHFFFAOYSA-N	3088497786	3062942132	3085604134	0.125170066
Mol 112	Sarmentosin epoxide	$C_{11}H_{17}NO_8$	291.09542	290.08834	2.209	Organic oxygen compounds	Organooxygen compounds	C(C1C(C(C(C(O1)OCC2C(O2)(CO)C#N)O)O)O)O	MGWCXJDKHMCXRL-YMGPVYFXSA-N	3094785098	3030228710	2903974235	0.122350724
Mol 113	2-Hydroxy-3-phenylpropenoate	$C_9H_8O_3$	164.04735	165.05484	2.496	Benzenoids	Benzene and substituted derivatives	C1=CC=C(C=C1)C=C(C(=O)O)O	DEDGUGJNLNLJSR-VURMDHGXSA-N	2520724665	3132129282	3242759851	0.120543386
Mol 114	3'-Methoxyflavonol	$C_{16}H_{12}O_4$	268.07356	269.08096	6.388	Phenylpropanoids and polyketides	Flavonoids	COC1=CC=CC(=C1)C2=C(C(=O)C3=CC=CC=C3O2)O	GYLGASXCHFNKHD-UHFFFAOYSA-N	2770570954	2833790173	3233462685	0.11976028
Mol 115	Hesperidin	$C_{28}H_{34}O_{15}$	610.18978	609.18292	5.668	Phenylpropanoids and polyketides	Flavonoids	COC1=CC=C(C=C1O)[C@@H]1CC(=O)C2=C(O1)C=C(OC1O[C@H](CO[C@H]3O[C@@H]([illegible]	QUQPHWDTPGMPEX-UNZJSITISA-N	2817083871	3269894085	2683047316	0.118841551
Mol 116	D-Allose	$C_6H_{12}O_6$	180.06339	203.05283	1.394	Organic oxygen compounds	Organooxygen compounds	C(C(C(C(C(C=O)O)O)O)O)O	WQZGKKKJIJFFOK-IVMDWMLBSA-N	2786716063	3333821816	2594462395	0.118095914
Mol 117	9(S)-HpOTrE	$C_{18}H_{30}O_4$	310.21441	309.20735	7.063	Lipids and lipid-like molecules	Fatty Acyls	CCC=CCC=CC=CC(CCCCCCCC(=O)O)OO	RWKJTIHNYSIIHW-MEBVTJQTSA-N	2794469419	2991437426	2737114884	0.115494436
Mol 118	(3S,5S)-Carbapenam-3-carboxylic acid	$C_7H_9NO_3$	155.05824	156.06565	1.979	Organoheterocyclic compounds	Lactams	C1CC(N2C1CC2=O)C(=O)O	RJPDELAUUYAFTQ-WHFBIAKZSA-N	2299541909	3071489075	3110571369	0.114933167
Mol 119	Hyperoside	$C_{21}H_{20}O_{12}$	464.09548	463.08867	5.643	Phenylpropanoids and polyketides	Flavonoids	OC[C@H]1O[C@@H](OC2=C(OC3=C(C(O)=CC(O)=C3)C2=O)C2=CC(O)=C(O)C=C2)[illegible]	OVSQVDMCBVZWGM-DTGCRPNFSA-N	2666043934	3149947988	2559469723	0.113494867
Mol 120	N-(Acetyloxy)benzenamine	$C_8H_9NO_2$	151.06333	152.07076	2.189	Organic nitrogen compounds	Organonitrogen compounds	CC(=O)ONC1=CC=CC=C1	BJKZXRGDVGYJTP-UHFFFAOYSA-N	2772958600	2758932607	2780909954	0.112645762

Mol 121	5-Desmethylsinensetin	$C_{19}H_{18}O_7$	358.10525	359.11266	6.414	Phenylpropanoids and polyketides	Flavonoids	COC1=C(C=C(C=C1)C2=CC(=O)C3=C(C(=C(C=C3O2)OC)OC)O)OC	QEWSAPKRFOFQIU-UHFFFAOYSA-N	3909552727	2021229459	2381551465	0.112639427
Mol 122	Maleic acid	$C_4H_4O_4$	116.01096	115.00356	1.936	Organic acids and derivatives	Carboxylic acids and derivatives	OC(=O)\C=C/C(O)=O	VZCYOOQTPOCHFL-UPHRSURJSA-N	2337659760	2955086343	2959390677	0.111823706
Mol 123	D-Pinitol	$C_7H_{14}O_6$	194.07904	217.06851	9.731	Organic oxygen compounds	Organooxygen compounds	CO[C@@H]1[C@@H](O)[C@@H](O)[C@H](O)[C@H](O)[C@H]1O	DSCFFEYYQKSRSV-KLJZZCKASA-N	2336784782	2888501246	2917887780	0.110347162
Mol 124	Gallicynoic acid F	$C_{18}H_{32}O_6$	344.21989	343.21264	6.033	Lipids and lipid-like molecules	Fatty Acyls	CCCCC(C#CC(C(C(CCCCCCCC(=O)O)O)O)O)O	DTCUTCQYZMBUDK-UHFFFAOYSA-N	2124197845	3992957294	1947010609	0.109276534
Mol 125	1,8-Diazacyclotetradecane-2,9-dione	$C_{12}H_{22}N_2O_2$	226.16813	227.17564	5.352	Phenylpropanoids and polyketides	Macrolactams	C1CCC(=O)NCCCCCCC(=O)NCC1	HERSSAVMHCMYSQ-UHFFFAOYSA-N	2632784328	2704447706	2600419232	0.107562151
Mol 126	N-(2,3-Dihydroxybenzoyl)-L-serine	$C_{10}H_{11}NO_6$	241.05864	240.05144	5.114	Benzenoids	Benzene and substituted derivatives	C1=CC(=C(C(=C1)O)O)C(=O)NC(CO)C(=O)O	VDTYHTVHFIIEIL-LURJTMIESA-N	2582370073	2636239585	2594575758	0.10587553
Mol 127	Gabaculine	$C_7H_9NO_2$	139.06333	140.07075	1.659	Organic acids and derivatives	Carboxylic acids and derivatives	C1C(C=CC=C1C(=O)O)N	KFNRJXCQEJIBER-UHFFFAOYSA-N	2772111843	2264389498	2659117599	0.104282401
Mol 128	2-Hydroxy-6-ketononatrienedioate	$C_9H_8O_6$	212.03209	211.02474	5.129	Organic acids and derivatives	Keto acids and derivatives	C(=CC(=O)C=CC(=O)O)C=C(C(=O)O)O	WCJYZUFKKTYNLB-ARITWGJRSA-N	2520212945	2775250802	2393027598	0.104185815
Mol 129	2-O-(alpha-D-Mannosyl)-D-glycerate	$C_9H_{16}O_9$	268.07943	267.07217	1.546	Lipids and lipid-like molecules	Fatty Acyls	C(C1C(C(C(C(O1)OC(CO)C(=O)O)O)O)O)O	DDXCFDOPXBPUJC-SAYMMRJXSA-N	2524714802	2701842026	2427940974	0.103725173
Mol 130	D-Fructose	$C_6H_{12}O_6$	180.06339	225.06158	1.382	Organic oxygen compounds	Organooxygen compounds	OC[C@H]1O[C@](O)(CO)[C@@H](O)[C@@H]1O	RFSUNEUAIZKAJO-RQDHWQXSA-N	2499007989	2701420200	2440790270	0.103545226

Mol 131	α,4-Dimethylstyrene	$C_{10}H_{12}$	132.0939	133.10123	6.147	Benzenoids	Benzene and substituted derivatives	CC1=CC=C(C=C1)C(=C)C	MMSLOZQEMPDGPI-UHFFFAOYSA-N	2477735732	2802864084	2326667417	0.103085157

Table S2. Score of 144 targets via CytoNCA.

Tgrget	Betweenness centrality (BC)	Closeness centrality (CC)	Degree centrality (DC)	Eigenvector centrality (EC)	Local average connectivity-based method (LAC)	Network centrality (NC)
AKT1	902.9317955727829	0.7566137566137566	97	0.16746185719966888	38.144329896907216	86.16969172696696
TNF	1069.9324987248563	0.7447916666666666	95	0.16367948055267334	37.38947368421053	83.61742461508125
PPARG	1072.2010442427886	0.7333333333333333	91	0.1571745127439499	36.13186813186813	77.28835936122393
TP53	478.47549857683146	0.715	88	0.16297796368598938	40.18181818181818	79.09695822435025
IL1B	692.7757921485381	0.7185929648241206	88	0.15712253749370575	37.34090909090909	75.0575291576504
SRC	1085.752704219769	0.7044334975369458	84	0.14950981736183167	35.714285714285715	66.17163072991984
STAT3	359.57715150960905	0.697560975609756	83	0.15933021903038025	40.626506024096386	72.05010045974998
JUN	256.8344022158948	0.6875	80	0.15686127543449402	40.95	69.10553151241656
BCL2	376.63248596475296	0.6908212560386473	80	0.15697282552719116	41	69.40892165741559
ESR1	532.5388353549043	0.6875	80	0.15136604011058807	38.525	65.72910009332456
CASP3	194.94082200825844	0.680952380952381	78	0.15636374056339264	41.84615384615385	68.18547770753241
PTGS2	430.96782104608894	0.680952380952381	77	0.14821209013462067	38.18181818181818	62.32901108936726
CTNNB1	406.65734286717367	0.680952380952381	77	0.14870886504650116	38.72727272727273	64.27356359190368
HIF1A	203.7095965326265	0.6651162790697674	74	0.14925117790699005	40.54054054054054	63.27797716710954
HSP90AA1	421.32052345367015	0.6651162790697674	72	0.13659881055355072	35.111111111111114	56.4844904216108
EGFR	219.26447613655608	0.6589861751152074	71	0.14308741688728333	39.098591549295776	58.267723255764935
MAPK3	237.4861493577424	0.6589861751152074	70	0.13966573774814606	37.22857142857143	55.255179566313906
MMP9	187.96145149584552	0.6559633027522935	70	0.14463664591312408	40.42857142857143	58.79601412001486
FOS	338.97380377490623	0.6529680365296804	68	0.13584771752357483	37.11764705882353	52.623998641304155
GSK3B	214.7120745472154	0.6470588235294118	67	0.13559824228286743	37.1044776119403	52.51898513845943

MTOR	146.87324636494193	0.6412556053811659	67	0.14032219350337982	40	56.73686802641685
CREB1	255.34799106842485	0.65	67	0.13089656829833984	35.1044776119403	50.186743928861915
BDNF	390.09023140937916	0.6470588235294118	65	0.1213693767786026	32.184615384615384	47.363369008961776
CCL2	232.85369697691203	0.6299559471365639	63	0.12358646094799042	33.523809523809526	49.495976400757506
PRKACA	452.8606172364106	0.6299559471365639	59	0.10614285618066788	26.135593220338983	37.5737598803846
CCND1	49.348570107830795	0.6137339055793991	57	0.12736181914806366	39.6140350877193	48.63300987942765
PPARA	227.81113096061276	0.6163793103448276	57	0.11337944120168686	31.017543859649123	41.88538202085046
MMP2	49.17846380964993	0.6111111111111112	55	0.12499870359897614	38.50909090909091	45.29899715964341
ERBB2	74.52150306680748	0.6111111111111112	54	0.117738276720047	35.03703703703704	42.22248411270388
IGF1R	37.23734453511652	0.6033755274261603	52	0.11861252784729004	37.07692307692308	42.488320598351464
JAK2	154.31016776427205	0.6008403361344538	52	0.11466605961322784	34.26923076923077	40.1951308076257
KDR	34.05085534215047	0.5983263598326359	51	0.11758774518966675	36.78431372549019	41.285041148861964
EDN1	313.88708047574966	0.6008403361344538	51	0.10041103512048721	27.49019607843137	34.29513704008327
NOS3	132.12580203096724	0.6008403361344538	50	0.10286930948495865	30.12	37.82190727574801
SMAD3	31.876697378317324	0.5933609958506224	49	0.11420110613107681	36.16326530612245	40.21506685752903
APP	274.45988471557575	0.5983263598326359	48	0.09881041198968887	27.583333333333332	33.007496127919694
MAPK1	64.39128056361658	0.5933609958506224	48	0.10839845985174179	33	37.52799404411867
ESR2	103.89730934866127	0.5933609958506224	47	0.10603822767734528	32.42553191489362	35.91623010864565
SLC2A4	184.74048847802644	0.588477366255144	46	0.08987895399332047	24.391304347826086	32.363385590227175
AR	325.1137153298651	0.5836734693877551	46	0.10583866387605667	33.608695652173914	36.75251845985061
MAPK8	19.739798441782444	0.5813008130081301	45	0.10742611438035965	34.75555555555555	37.42190209811231
SOX2	38.495058477007895	0.5813008130081301	44	0.1025729775428772	33.22727272727273	36.09473534495179
CDK2	82.10157313173754	0.5789473684210527	44	0.09546047449111938	28.318181818181817	32.393306907199786
MAOA	436.33152643386825	0.5836734693877551	43	0.05767408758401871	15.581395348837209	24.954110815827427

PIK3CA	15.081720970560719	0.572	42	0.09813017398118973	32.95238095238095	34.91530733438629
MAPK14	30.488065575935682	0.5674603174603174	41	0.09868651628494263	31.5609756097561	33.47866045973765
PIK3R1	21.482512200731858	0.5697211155378487	41	0.09423355013132095	29.902439024390244	31.902330418959277
KIT	40.13643106221229	0.5742971887550201	40	0.09379713237285614	29.6	31.545637973042705
MET	22.118142163783443	0.5674603174603174	40	0.09464399516582489	30.75	32.26492341507822
TH	230.331915681727	0.5766129032258065	40	0.07064152508974075	19.9	25.00052802896732
CD36	177.21369470306996	0.5697211155378487	40	0.07744596153497696	21.8	27.122879027341273
CYBB	30.15407637353221	0.572	40	0.08659442514181137	28.05	31.787097004480383
SLC2A1	47.36395473923796	0.5697211155378487	39	0.09008436650037766	27.487179487179485	29.988064337909535
CDK4	24.524026323014944	0.5697211155378487	39	0.09209825843572617	30.307692307692307	32.264480969202324
ABCB1	109.94324278341477	0.5674603174603174	39	0.07997062802314758	22.564102564102566	25.84136062807486
MPO	57.02596369240992	0.5652173913043478	38	0.07817422598600388	24.473684210526315	28.67190364885092
CCNA2	32.249997096384924	0.5607843137254902	38	0.08855830132961273	28.894736842105264	31.525917602308294
PTPN1	80.9382414689003	0.5607843137254902	36	0.07728125154972076	22.22222222222222	24.13648551461685
XIAP	52.747675547981196	0.556420233463035	35	0.0827617198228836	26.514285714285716	28.422619047619047
ADRB2	330.64853109733247	0.562992125984252	35	0.05620498210191727	14.457142857142857	19.145828164210513
IKBKB	11.328405765870757	0.5542635658914729	35	0.0852607935667038	27.485714285714284	28.749605589362176
NOX4	14.69122659221903	0.556420233463035	34	0.07935088127851486	26.058823529411764	27.988553349587825
TERT	17.44131022808251	0.5521235521235521	34	0.0805291086435318	27.41176470588235	28.679875679875675
CDK1	20.784866458562767	0.5521235521235521	34	0.0792706161737442	25.823529411764707	27.73748896797898
NTRK2	72.42853136473471	0.5542635658914729	33	0.06412910670042038	18.78787878787879	20.88650238061316
CYP19A1	140.32505745429782	0.556420233463035	33	0.07384537905454636	21.454545454545453	23.020323426573427
DRD2	123.60766105495756	0.5478927203065134	32	0.04299628734588623	14.1875	18.82069391972144
XDH	49.613901257539005	0.5478927203065134	30	0.060518328100442886	19.2	21.435021240122655

NCOA1	43.506781690251124	0.5416666666666666	30	0.0629226490855217	18.733333333333334	21.172550509013856
MMP3	5.184871091986378	0.5458015267175572	30	0.07580031454563141	25.466666666666665	26.564263322884013
NOS2	16.5115888680698	0.5458015267175572	30	0.06757386773824692	21.933333333333334	23.354182050388946
OPRM1	67.18312612758953	0.5478927203065134	28	0.04799318313598633	14.785714285714286	16.66927979427979
INSR	36.740550647027966	0.5355805243445693	28	0.059424128383398056	17.642857142857142	19.18714055670577
NLRP3	6.816373165919322	0.539622641509434	28	0.06678958237171173	22.857142857142858	23.990067340067338
ARG1	16.008922676849814	0.539622641509434	28	0.06568487733602524	21.571428571428573	23.17592592592592
PIK3CG	3.4482412180725786	0.5375939849624061	28	0.07167892158031464	23.857142857142858	24.740740740740737
SLC6A4	110.18320610496677	0.5335820895522388	27	0.02970193326473236	12.222222222222221	16.010461515608576
CYP1A1	35.86759678634538	0.5315985130111525	27	0.056701384484767914	17.333333333333332	19.63938883389684
DPP4	64.44194365132297	0.5375939849624061	27	0.05149073526263237	14.074074074074074	15.836673343706593
SLC6A3	99.06036906590946	0.5355805243445693	27	0.028760485351085663	11.925925925925926	15.348665631753867
TYR	40.05070699584286	0.5416666666666666	27	0.05497291311621666	14.592592592592593	15.665981077745782
GSR	42.15816764192992	0.5355805243445693	26	0.05553271621465683	17.615384615384617	18.779411764705884
NR1H4	53.12792209220354	0.5276752767527675	24	0.043952587991952896	11.75	13.463216892588276
CYP1A2	86.66938226501266	0.5276752767527675	23	0.031003128737211227	8.434782608695652	9.950026066125137
MMP13	2.7060221931897828	0.5218978102189781	23	0.05942007154226303	19.91304347826087	20.818181818181817
HTR1A	34.958541498233224	0.5296296296296297	23	0.029389848932623863	12.869565217391305	14.173929420252946
DRD1	50.447993765692	0.5143884892086331	22	0.02630596235394478	11.272727272727273	13.16829836829837
HTR2A	67.61718811904771	0.52	22	0.023717600852251053	9.636363636363637	10.908064892623718
SLC6A2	74.49949866523579	0.49480968858131485	22	0.01798284612596035	8.545454545454545	10.984340985811572
RXRA	18.50836534660185	0.5238095238095238	22	0.04664401337504387	14.545454545454545	16.035087719298247
SIRT3	5.830611560309802	0.5088967971530249	21	0.050829678773880005	17.142857142857142	18.249999999999996
NR1I2	42.81099512174266	0.5107142857142857	20	0.03229234367609024	9.5	10.642363484468747

CDK5	2.3864547096994797	0.5107142857142857	20	0.048654716461896896	16.2	17.052631578947366
CYP2D6	46.45804910414759	0.4965277777777778	19	0.015567581169307232	8.105263157894736	8.822245564892624
CYBA	2.9502815115051706	0.516245487364621	19	0.04387703910470009	15.263157894736842	16.111111111111114
F3	39.954464427305815	0.5107142857142857	19	0.039066094905138016	11.789473684210526	13.461538461538462
CFTR	130.18526394162907	0.5238095238095238	18	0.028435874730348587	7	9.007609710550888
BCHE	44.06742595646422	0.5053003533568905	18	0.028532162308692932	7.888888888888889	9.017659627953746
HTR2C	30.159875033334274	0.4880546075085324	18	0.013289414346218109	8.777777777777779	9.96328377504848
AKR1B1	63.30206835972882	0.5181159420289855	18	0.03234254568815231	10.11111111111111	11.844444444444447
ADRB1	41.90877771662015	0.5125448028673835	17	0.01868712715804577	6.352941176470588	7.242582417582418
SIGMAR1	298.5258324705981	0.5143884892086331	17	0.02188023552298546	8.705882352941176	9.276785714285715
FAS	7.086146638974696	0.5088967971530249	17	0.044403690844774246	14.235294117647058	15.366071428571429
PGK1	13.477981683001243	0.49140893470790376	16	0.03217529505491257	9.125	10.11111111111111
KCNH2	55.48384953345633	0.5107142857142857	15	0.014867972582578659	5.066666666666666	5.9
ADRB3	19.440300095265975	0.5107142857142857	15	0.027245931327342987	8.666666666666666	9.333333333333332
ADCY5	41.21113754582348	0.4965277777777778	14	0.015347330830991268	5	5.653346653346654
DRD4	14.657704301275102	0.48639455782312924	14	0.015312612056732178	7.428571428571429	8.141025641025642
ALOX5	5.738053012580104	0.5	14	0.03393039107322693	10.857142857142858	11.890109890109894
F2	41.358779385622356	0.4880546075085324	14	0.01970691792666912	4.714285714285714	6.032051282051283
ADRA2A	12.035874954491074	0.4735099337748344	13	0.010362689383327961	7.076923076923077	7.872727272727273
PDE5A	6.23603449139053	0.49480968858131485	13	0.026347825303673744	8.461538461538462	9.166666666666668
AVPR2	31.81352617965796	0.4847457627118644	13	0.014307016506791115	4.923076923076923	5.413636363636364
CYP1B1	5.757533230036745	0.4766666666666667	12	0.020426422357559204	7.333333333333333	8.121212121212121
ADRA2C	12.791491389495501	0.4798657718120805	12	0.011224857531487942	5.5	6.036363636363635
ADRA2B	11.405298331604286	0.45686900958466453	12	0.007007251493632793	6.166666666666667	6.827272727272726

SLC5A2	10.432349609601529	0.5035211267605634	12	0.024861296638846397	7.833333333333333	8.545454545454545
ADORA1	19.893887869594565	0.5	11	0.016868315637111664	4.545454545454546	5
ADRA1A	9.335154531703166	0.43202416918429004	11	0.006800290197134018	5.454545454545454	5.999999999999999
ADRA1B	15.040791200571988	0.4454828660436137	11	0.007094140164554119	4	4.4
SIRT2	1.5415061266544139	0.4766666666666667	11	0.025850526988506317	8.363636363636363	9.2
PRKAA1	3.2346136288177174	0.4798657718120805	11	0.022222377359867096	7.2727272727272725	8.088888888888889
AHR	3.6037719737870755	0.4583333333333333	10	0.016564175486564636	6.4	7.111111111111111
PYGL	10.77414528755209	0.4750830564784053	10	0.0174129456281662	4.4	5.111111111111111
TTR	4.664312438024734	0.4766666666666667	10	0.01927158236503601	5.4	5.999999999999999
PRKAA2	2.156135486072568	0.4798657718120805	10	0.020613009110093117	7.2	8.000000000000002
ADA	15.934644634066036	0.4612903225806452	9	0.013867704197764397	3.7777777777777777	4.25
MERTK	0.8011399711399712	0.4750830564784053	9	0.02093520574271679	6.666666666666667	7.5
ABCC8	10.279487627473994	0.4782608695652174	8	0.011256376281380653	2.5	2.971428571428571
NEK2	0.23687270214529033	0.47039473684210525	8	0.01959935575723648	6.25	7.142857142857142
AOC3	2.887943423491024	0.47194719471947194	8	0.01461431011557579	4.5	5.142857142857142
CA2	27.66466125296948	0.4612903225806452	7	0.011888504028320312	2.2857142857142856	3
KCNMA1	3.501099541958684	0.46579804560260585	6	0.00940009020268917	2.6666666666666665	3.1999999999999997
NR1H2	0.09523809523809523	0.4583333333333333	6	0.011188549920916557	4.666666666666667	5.6
ACP1	3.2563499887901832	0.4525316455696203	5	0.008650430478155613	1.2	1.5
CFD	0	0.4642857142857143	5	0.011894631199538708	4	5
CTSC	0.27705627705627706	0.44135802469135804	4	0.008717378601431847	2	2.6666666666666665
PNLIP	0.6775379570639977	0.43465045592705165	4	0.007346549071371555	2	2.6666666666666665
SCNN1A	0.7715909090909091	0.3753280839895013	3	0.0018768857698887587	1.3333333333333333	2
QDPR	0	0.3896457765667575	3	0.0031237227376550436	2	3

SOAT1	0.2490842490842491	0.41329479768786126	3	0.004862906411290169	0.6666666666666666	1
F7	0	0.43465045592705165	3	0.0043251425959169865	2	3
AKR1C2	0	0.3695090439276486	1	0.0020579544361680746	0	0
EBP	0	0.3404761904761905	1	4.254264058545232E-4	0	0
**Median value**	40.6737843	0.541666667	28	0.0594221	18.18809524	20.8523421

Table S3. Score of 48 targets via CytoNCA.

Tgrget	Betweenness centrality (BC)	Closeness centrality (CC)	Degree centrality (DC)	Eigenvector centrality (EC)	Local average connectivity-based method (LAC)	Network centrality (NC)
JUN	25.872364644207874	0.9791666666666666	46	0.17331412434577942	34.30434782608695	45.4240297951742
ERBB2	8.726082773939039	0.7833333333333333	34	0.13435353338718414	28	30.833017086257648
APP	4.791657671186058	0.7580645161290323	32	0.13108202815055847	27.9375	29.75687563027297
XIAP	0.7305340102308064	0.6811594202898551	25	0.10565852373838425	23.28	24.329545454545453
TP53	25.431722855282324	0.9791666666666666	46	0.17355726659297943	34.391304347826086	45.46052214632245
ESR2	4.987214274891212	0.7833333333333333	34	0.13860967755317688	29.58823529411765	31.535270840205975
AKT1	23.81360944519591	0.9591836734693877	45	0.17023146152496338	33.77777777777778	43.716620071270704
PTGS2	21.75735723122631	0.9591836734693877	45	0.1715337038040161	34.4	44.19745578280163
STAT3	21.447417781271373	0.9591836734693877	45	0.17157652974128723	34.4	44.15200913683253
MTOR	14.098097027391946	0.8867924528301887	41	0.15997189283370972	32.829268292682926	38.73727810197509
AR	1.9087674391489442	0.746031746031746	31	0.12983223795890808	28.387096774193548	29.758333333333326
HIF1A	16.638073831821085	0.9215686274509803	43	0.16654875874519348	34	41.55967370037309
CDK2	0.9690963341858481	0.6811594202898551	25	0.10617392510175705	23.12	24.11413043478261
MAPK3	21.126472645419945	0.9215686274509803	43	0.16334925591945648	32.51162790697674	40.51631414548828
XDH	0.09090909090909091	0.618421052631579	18	0.07646580040454865	16.88888888888889	17.88235294117647
SLC2A1	2.570089857761862	0.7230769230769231	29	0.12086108326911926	26	27.326479076479078
PRKACA	5.82722943516953	0.7833333333333333	34	0.1370849460363388	29	30.931494580352606
CCL2	7.159222805961343	0.8103448275862069	36	0.14501598477363586	30.72222222222222	33.56755370525532
PPARA	9.203565602092738	0.8103448275862069	36	0.14353615045547485	29.944444444444443	33.04347718711309
BDNF	9.069070541891314	0.8245614035087719	37	0.14713570475578308	30.81081081081081	34.2993174692209

ABCB1	0.2007385730211817	0.6619718309859155	23	0.09815116971731186	21.73913043478261	22.72727272727273
CREB1	10.018095702619792	0.8545454545454545	39	0.15479028224945068	32.46153846153846	36.812588566180736
ESR1	25.617917102174516	0.9791666666666666	46	0.17346473038196564	34.34782608695652	45.430296813941695
HSP90AA1	8.558321498554438	0.8392857142857143	38	0.1517956554889679	31.94736842105263	35.68457650675393
CYP19A1	0.9696761359272061	0.6714285714285714	24	0.10100658982992172	21.916666666666668	22.869565217391305
TNF	28.489203764466023	1	47	0.17592822015285492	34.5531914893617	47
NTRK2	1.4073557218080543	0.6351351351351351	20	0.08275946974754333	17.6	18.526315789473685
GSK3B	16.50703076535434	0.9038461538461539	42	0.16207613050937653	32.904761904761905	39.796464113942235
PPARG	20.01314455311607	0.94	44	0.16850948333740234	34	42.73242969563769
CCND1	5.990904219547259	0.8103448275862069	36	0.14529390633106232	31.11111111111111	33.99456810137035
TH	1.2466780237464552	0.6527777777777778	22	0.09154264628887177	19.727272727272727	20.73182957393484
CD36	1.364133241112646	0.6619718309859155	23	0.09502212703227997	20.608695652173914	21.545454545454543
MMP2	10.514189822327639	0.8703703703703703	40	0.15855103731155396	33.05	37.87052523100453
MAPK1	3.640643696568494	0.7580645161290323	32	0.1323961764574051	28.5	30.024116743471584
BCL2	25.3188552876934	0.9791666666666666	46	0.1736769676208496	34.43478260869565	45.4814957054539
IL1B	25.69027270195979	0.9791666666666666	46	0.1733877807855606	34.34782608695652	45.460567966055756
JAK2	5.113159372298742	0.7580645161290323	32	0.1308588683605194	27.6875	29.526771218496325
CASP3	28.489203764466023	1	47	0.17592822015285492	34.5531914893617	47
SLC2A4	3.132585432956799	0.7121212121212122	28	0.11511971056461334	24.571428571428573	25.809932659932656
MPO	1.8922081742733916	0.6714285714285714	24	0.09855283051729202	21.416666666666668	22.624970936991396
FOS	11.50083476285298	0.8703703703703703	40	0.1578439623117447	32.9	37.90869489362241
CTNNB1	17.245933466864052	0.9215686274509803	43	0.16594667732715607	33.76744186046512	41.43798536112727
SRC	18.659308491516846	0.9215686274509803	43	0.1650925874710083	33.30232558139535	41.05329802047917
EDN1	3.033600935304917	0.734375	30	0.12382960319519043	26.866666666666667	28.44300072186129
NOS3	5.102039166071522	0.7704918032786885	33	0.13381639122962952	28.727272727272727	30.897045296592502

MMP9	16.638073831821085	0.9215686274509803	43	0.16654875874519348	34	41.5596737003731
EGFR	13.613898564587346	0.8867924528301887	41	0.1601070761680603	33.02439024390244	38.97847104758146
PTPN1	1.813437927804448	0.6438356164383562	21	0.08690572530031204	18.095238095238095	19.031578947368423
**Median value**	8.897576658	0.817453116	36.5	0.146214806	30.96096096	34.14694279

Table S4. Score of 20 targets via CytoNCA.

Tgrget	Betweenness centrality (BC)	Closeness centrality (CC)	Degree centrality (DC)	Eigenvector centrality (EC)	Local average connectivity-based method (LAC)	Network centrality (NC)
TNF	28.48920376	1	47	0.17592822	34.55319149	47
CASP3	28.48920376	1	47	0.17592822	34.55319149	47
JUN	25.87236464	0.979166667	46	0.173314124	34.30434783	45.4240298
IL1B	25.6902727	0.979166667	46	0.173387781	34.34782609	45.46056797
ESR1	25.6179171	0.979166667	46	0.17346473	34.34782609	45.43029681
TP53	25.43172286	0.979166667	46	0.173557267	34.39130435	45.46052215
BCL2	25.31885529	0.979166667	46	0.173676968	34.43478261	45.48149571
AKT1	23.81360945	0.959183673	45	0.170231462	33.77777778	43.71662007
PTGS2	21.75735723	0.959183673	45	0.171533704	34.4	44.19745578
STAT3	21.44741778	0.959183673	45	0.17157653	34.4	44.15200914
PPARG	20.01314455	0.94	44	0.168509483	34	42.7324297
SRC	18.65930849	0.921568627	43	0.165092587	33.30232558	41.05329802
CTNNB1	17.24593347	0.921568627	43	0.165946677	33.76744186	41.43798536
HIF1A	16.63807383	0.921568627	43	0.166548759	34	41.5596737
MMP9	16.63807383	0.921568627	43	0.166548759	34	41.5596737
MTOR	14.09809703	0.886792453	41	0.159971893	32.82926829	38.7372781
EGFR	13.61389856	0.886792453	41	0.160107076	33.02439024	38.97847105
FOS	11.50083476	0.87037037	40	0.157843962	32.9	37.90869489
MMP2	10.51418982	0.87037037	40	0.158551037	33.05	37.87052523
CREB1	10.0180957	0.854545455	39	0.154790282	32.46153846	36.81258857
